# Biological outcome and mapping of total factor cascades in response to HIF induction during regenerative angiogenesis

**DOI:** 10.18632/oncotarget.7728

**Published:** 2016-02-25

**Authors:** Abdel-Majid Khatib, Rachid Lahlil, Martin Hagedorn, Claudine Delomenie, Olivier Christophe, Cecile Denis, Geraldine Siegfried

**Affiliations:** ^1^ Université Bordeaux, Pessac, France; ^2^ INSERM, LAMC, UMR 1029, Pessac, France; ^3^ INSERM U 770, Kremlin-Bicêtre, France; ^4^ IFR141 IPSIT, Université Paris Sud, Chatenay-Malabry, France

**Keywords:** HIF, CoCl2, angiogenesis, regeneration, gene ontology, functional enrichment, Pathology Section

## Abstract

Hypoxia Inducible Factor (HIF) is the main transcription factor that mediates cell response to hypoxia. Howeverthe complex factor cascades induced by HIF during regenerative angiogenesis are currently incompletely mapped and the biological outcome mediated by chronic HIF induction during vessel regeneration are not well known. Here, we investigated the biological impact of HIF induction on vascular regeneration and identified the differentially regulated genes during regeneration, HIF induction and hypoxic regeneration. The use of the fin zebrafish regeneration model revealed that exposure to HIF inducer (cobalt chloride) prevents vessel differentiation by maintaining their vascular plexuses in an immature state. The regenerated fins are easily breakable, lacking completely endochondral ossification. Gene expression arrays combined to gene functional enrichment analysis revealed that regenerative process and HIF induction shared the regulation of common genes mainly involved in DNA replication and proteasome complex. HIF induction during regeneration affected the expression of exclusive genes involved in cell differentiation and communication, consistent with the observed immature vascular plexuses of the regenerated fins during HIF induction. The use of morpholino (MO) knockdown strategy revealed that the expression of some of these genes such as tubulin and col10a1 are required for fin regeneration. Taken together, this study revealed the impact of HIF induction on regenerative angiogenesis and provided a framework to develop a gene network leading to regenerative process during HIF expression.

## INTRODUCTION

The need of angiogenesis is important during embryogenesis, wound healing, remodeling of ischemic muscle and fracture repair as well as in pathological situations, including myocardial ischemia, ocular pathologies, and tumor growth [1-3]. Thus, the molecular and genetic dissection of angiogenic process leading to vessel regeneration under normal and perturbed situations is clinically relevant [4, 5]. Recently, focusing on inducing, rather than mimicking the angiogenic process through HIF/hypoxia emerged as a new field of angiogenesis research. This is due to the limited success of strategies that relied on exogenous delivery of angiogenic factors as a therapeutic approach. Indeed, previously, strategies in clinical trials highlighted the complexity and difficulty regarding the ability of isolated delivery of certain angiogenic factors to mimic the complete angiogenic response [1-6]. In contrast, HIF/hypoxia-induced signaling used as an endogenous tool seemed to mimic the natural biological mechanism responsible for the induction of angiogenesis in physiological as well as in pathological circumstances [5, 6]. Although, this approach has the advantage that it can be easily and effectively applied, and the derived knowledge can provide the identification and the development of more targeted therapies, the biological outcome and the side effects following chronic HIF induction is still not well known. Furthermore, the complex factor cascades induced by HIF during vessels regeneration remain uncharted area.

Like in various lower vertebrates several missing cells and tissues in adult zebrafish are faithfully replaced by regenerative process [7, 8]. In contrast, in humans and other mammals this regenerative capacity is very limited, but the mechanism seem to be conserved. Several genes such as *Max* and *BMP* were found to initiate and participate in the regeneration of mammalian digit tips [9, 10] and during zebrafish caudal fin regeneration [11-13]. Thereby regeneration studies using the zebrafish model have the potential to uncover important genes and pathways that have been inactivated in mammals [8, 14]. In addition, there is a high degree of conservation between zebrafish and mammals regarding the pathways involved in angiogenesis [15, 16] including the hypoxia-inducible factor (HIF) signaling pathways [17]. In the current study, we have taken advantage of the transgenic *TG (Fli1: EGFP)* zebrafish line [18], in which the vascular system is visible through endothelial-specific enhanced green fluorescent protein *(EGFP)* expression to examine the effect of HIF induction on regenerative angiogenesis. We revealed that under the influence of the HIF inducer CoCl_2_ fin vessel regeneration and remodeling were profoundly deregulated leading to perturbed vessel maturation and bone formation. The use of microarray analysis and functional enrichment analysis mapped all the biological functions linked to HIF induction, regeneration and to their combined effect. In addition, the use of synthetic morpholino antisense oligonucleotides (MOs) strategy revealed that some of the tested highly expressed genes are required for fin regeneration. The generated gene expression database used in the current study may also constitute a useful tool for strategies aiming to use HIF/hypoxia-based angiogenesis, now emerging as a new therapeutic approach.

## RESULTS

### Hypoxia-inducible transcription factor (HIF) and fin regeneration

**Figure 1:**
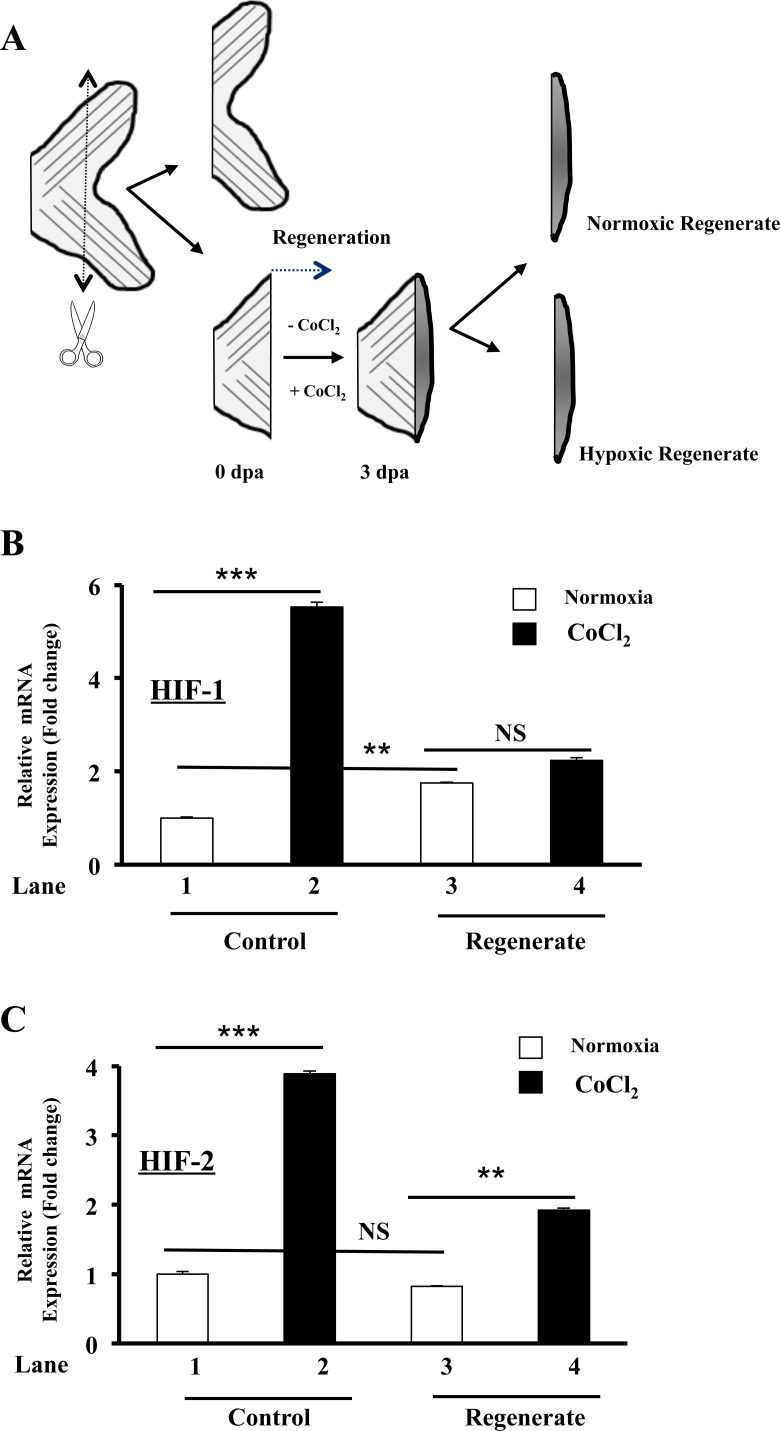
Regulation of HIF-1 and HIF-2 expression by CoCl2 during zebrafish fin regeneration **A.** Schematic representation of experimental plan showing amputation site and regenerated fin areas 3 days post amputation (3dpa) in the absence (−CoCl_2_) and presence (+ CoCl_2_) of CoCl_2_. Amputation was performed at a level proximal to the first bifurcation of the bony rays, and fins were allowed to regenerate prior analysis. **B.**-**C.** Fold change in HIF-1 and HIF-2 mRNA expression, as revealed by real time PCR analysis of total RNA obtained from control and regenerated fins exposed or not to CoCl_2_. Data shown represents Mean ± S.E (*n* = 15 per group) from three independent experiments. NS: not significant. **P <0.001. *** *P <*0.0001.

To evaluate the effect of HIF induction on regenerative angiogenesis, we first investigated the effect of CoCl_2_ on HIF-1 and HIF-2 expression during fin regeneration. 50 % of the caudal zebrafish fins were surgically removed, and fish were immediately placed in either water or water containing CoCl_2_ to allow regeneration (Figure 1A). The expression of HIF-1 (Figure 1B) and HIF-2 (Figure 1C) was monitored in control and regenerated fins, respectively. In controls, 3 days post-amputation (dpa), the expression of HIF-1 was increased by up to 2 fold (Figure 1B, Lanes 1, 3) and HIF-2 (Figure 1C, Lanes 1, 3) remained unchanged in the regenerated fin area. In the presence of CoCl_2_ the induction of HIF-1 and HIF-2 was increased by up to 5.5- (Figure 1B, Lanes 1, 2) and 4- (Figure 1C, Lanes 1, 2) fold, in control fins, respectively. In the regenerated area, HIF-1 expression didn't change significantly (Figure 1B, Lanes 3, 4) and HIF-2 expression remained upregulated as compared to control fins (Figure 1C, Lanes 1, 3, 4). These findings suggest that regenerative process seemed to attenuate HIF-1 and HIF-2 induction mediated by CoCl_2_.

### HIF induction and vessel formation during fin regeneration

**Figure 2:**
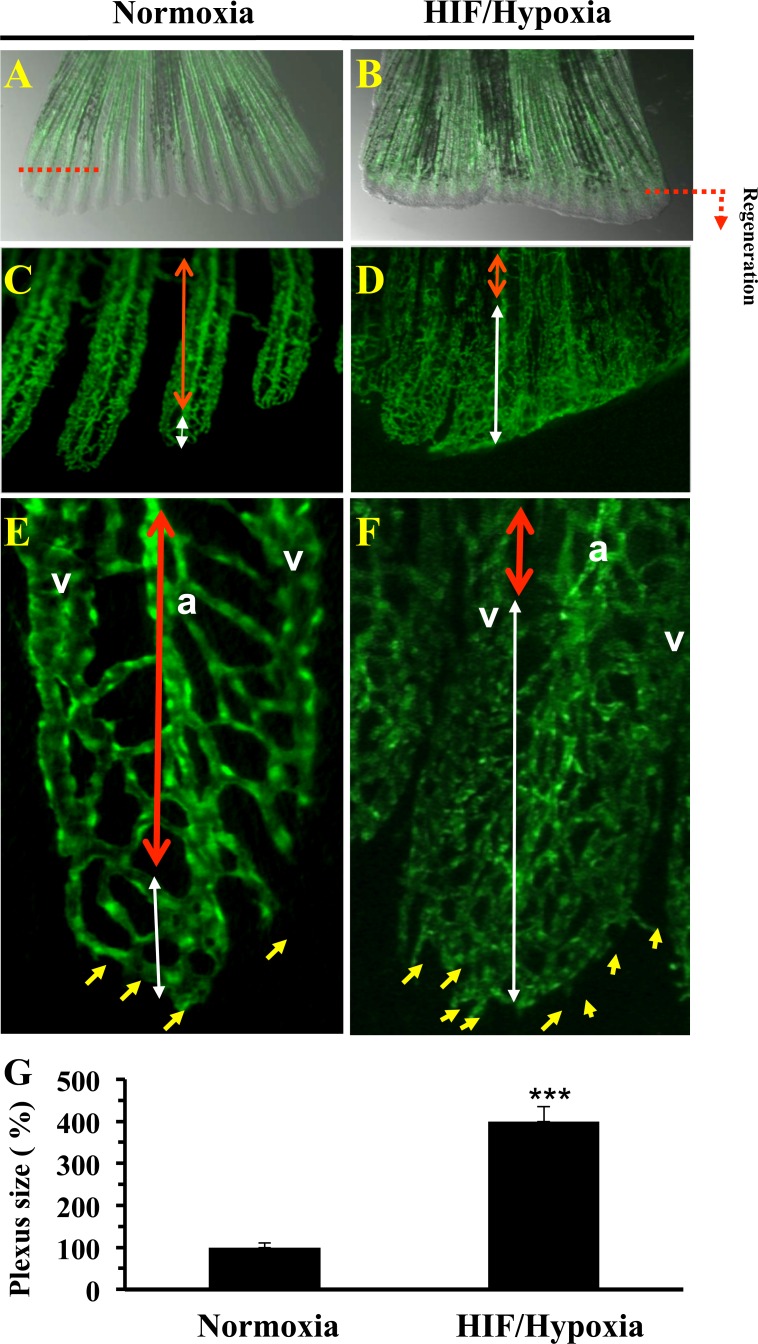
Vasculature plexus formation and remodeling during regenerative angiogenesis and HIF induction Vasculature of regenerated transgenic zebrafish *Tg (fli1: EGFP)* caudal fin under normoxic control conditions **A.** and following exposure to CoCl_2_ (1mM) **B.** In controls, by 3-6 dpa, the plexus is remodeled into distinguishable arteries (a) and veins (v) in the proximal regenerate (region delineated by red doubled-headed arrows) **C.**, **E.** A vasculature plexus is still present at distal end (region delineated by white doubled-headed arrows). E, F), Higher magnification of regenerated fins. **D.**, **F.** Following CoCl_2_ (1mM) exposure regenerates failed to complete plexus remodeling adequately. No clear distinguishable arteries and veins in the proximal area of the regenerate are observed (region delineated by blue doubled-headed arrows). Distal ends of regenerates treated with CoCl_2_ indicated increased sprouts number (yellow arrows) formed at the distal end of the growing vessel. **G.** Results of plexus length measurement are shown in the bar graph and are expressed as the percentage of length of none clear distinguishable arteries and veins structure relative to those observed under normoxia, which was assigned a value of 100%. Original magnification: 2-x objectives for A-B, 10-x objective for C-D, and 40-x objective for E-F. The amputation plane is indicated. Data shown represents Mean ± S.E (*n* =15 per group) from three independent experiments. *** *P <*0.0001.

Previously, formation of a transient vascular plexus was observed during blood vessel regeneration in zebrafish fins prior to complete fin regeneration [18, 22]. The formation of vascular plexus in regenerating fins appears only between 3 and 8 days post-amputation [18]. We thereby investigated the effect of HIF induction on vascular plexus formation and vessel regeneration. To this aim, we used the transgenic zebrafish *Tg (fli1: EGFP)* line that expresses EGFP in all endothelial cells, under the control of the zebrafish *fli1* promoter [18, 22]. Analysis of blood vessels in regenerated fins revealed that in controls, plexus formation was more limited. As can be seen in Figure 2A, 2C, 2E. A well ordered vascular structure is observed, as evidenced by a thickened central vessel with more intense EGFP fluorescence, which extends from the artery in the stump. At the tip of each fin ray a U-shaped connections were present (Figure 2E). In contrast, exposure to CoCl_2_ inhibited this process by up to 4 fold (Figure 2B, 2D, 2F, 2G). Blood vessels appear denser with the continuing formation of vessels at the distal tips of the regenerating fins (Figure 2F), indicating intense vessel formation and reduced vessel remodeling. There is no clear morphological distinction between arteries and veins in the regenerating blood vessel plexuses; suggesting a continuity of blood vessel regeneration. Thus, HIF induction seems to participate in the maintaining of immature vascular network in the regenerating fins. This phenomenon was associated with increased numbers of tip cells, indicators of undifferentiated endothelial cells [23] (Figure 2F).

### Regeneration of fins lacking bone differentiation during HIF induction

**Figure 3:**
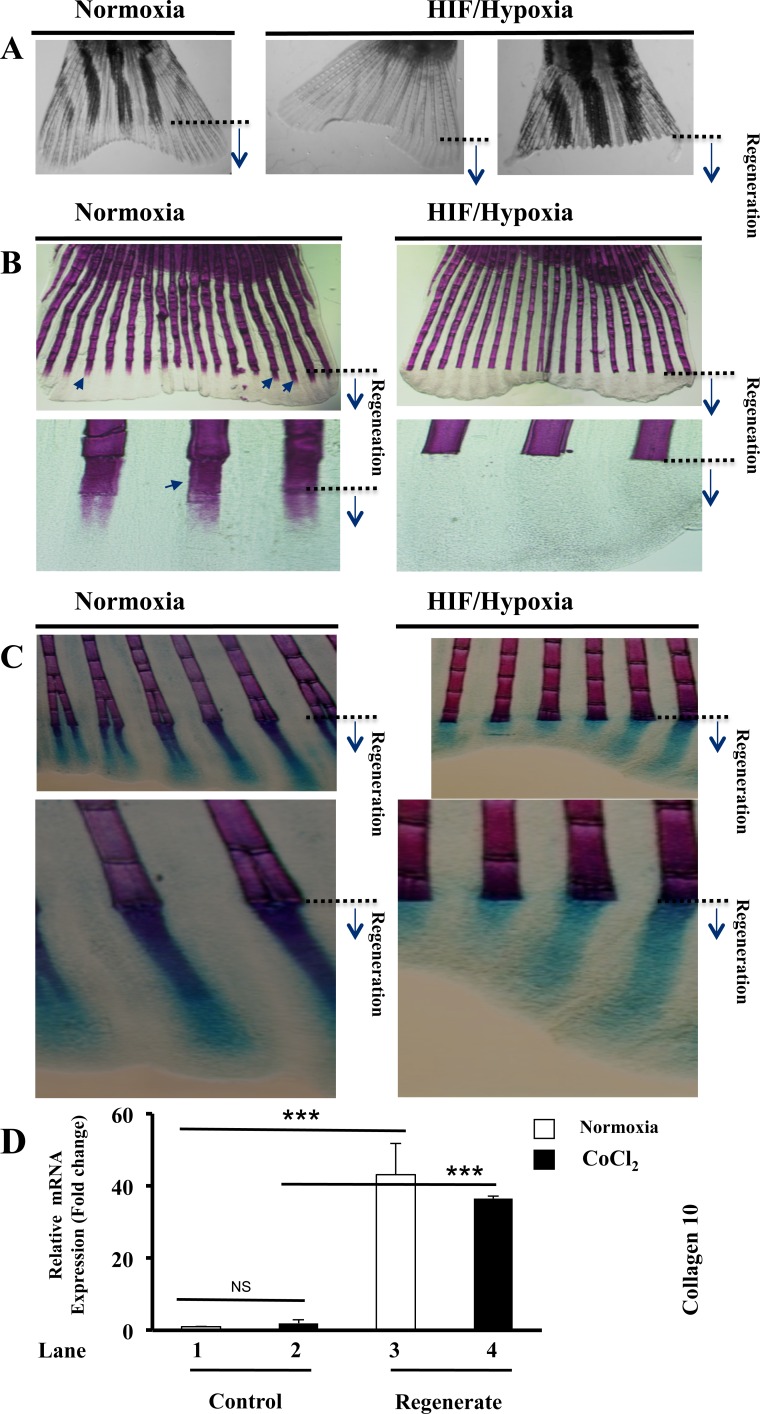
Maintained hypertrophic cartilage formation and inhibition of bone formation during HIF induction **A.** Fish exposed to CoCl_2_ after fin amputation; for 5-6 days, regenerated very fragile fins that fissure easily, leading to their partial or total loss, while they progress in water. **B.** Alizarin red-staining of fins exposed or not to CoCl_2_, shows staining of fin rays, including newly regenerated areas (black arrows). Fish exposure to CoCl_2_ induced loss of Alizarin red staining in regenerated fin areas. Note the inhibition of bone ossification behind the site of amputation in the presence of CoCl_2_. Original magnifications: 2-x objective for A, 10-x and 40-x objectives for B. **C.** Double staining of bone (Alizarin red) and cartilage (Alcian-blue) in regenerated fins exposed or not to CoCl_2_. Note that while exposure to CoCl_2_, induced loss of Alizarin red staining in the regenerated fin area, Alcian-blue staining pattern was well maintained throughout regenerated fin area. Original magnifications: 10-x and 40-x objective, respectively. **D.** Fold change in the expression of the hypertrophic marker collagen 10, as measured by real time PCR analysis of total RNA obtained from control and regenerated fins, exposed or not to CoCl_2_ (1mM). Note the highly induced expression of collagen 10 during regeneration (lanes 1 and 3) and its maintained high expression during hypoxia (lanes 2 and 4). Data shown represents Mean ± S.E (*n=*6 per group) from three independent experiments. ***P<0.0001.

Following exposure of fish to CoCl_2_ during fin regeneration we observed that regenerated fins are very fragile and can easily fissure leading to their partial or total loss while fish progress in water (Figure 3A). To determine whether HIF induction that mediates fin fragility is linked to a defect in fin skeleton, we tested whether bone forms normally in fins under these conditions. As illustrated in Figure 3B, in control conditions, Alizarin red staining for bone at 5 dap showed a strong calcification in the regenerated fins. Fin exposure to CoCl_2_ during the same period blocked completely this process, as revealed by the absence of red staining in the regenerates. Consistent with this finding while cartilage hypertrophy precedes endochondral ossification process, we also tested whether CoCl_2_ affect cartilage formation during fin regeneration. Alcian blue and Alizarin red double staining showed that while Alizarin staining is absent in the regenerates in the presence of CoCl_2_, the Alcian blue revealed the presence of cartilage in the regenerated fins, under normoxic and HIF/hypoxic conditions (Figure 3C). During endochondral ossification, immature chondrocytes enlarge to form hypertrophic chondrocytes, which express collagen 10 [23]. Expression analysis of this hypertrophic marker revealed that under normoxic conditions its expression was dramatically increased during regeneration (up to 40 fold) (Figure 3D, lanes 1, 3). In control fins, CoCl_2_ failed to induce significantly collagen 10 expression (Figure 3D, lanes 1, 2). In regenerating fins the expression of this marker remained significantly higher in the presence of CoCl_2_ (Figure 3D, Lanes 2, 3, 4). This finding suggests that HIF induction effectively inhibits the recruitment of osteoblasts from precursor cells still present in the regenerated fins. Thereby, in the presence of CoCl_2_, chondrocytes would not contribute to the formation and remodeling of bone, leading to the regeneration of fragile fins (Figure 3A).

### Analysis of differentially expressed genes during regenerative angiogenesis and HIF/Hypoxia

**Figure 4:**
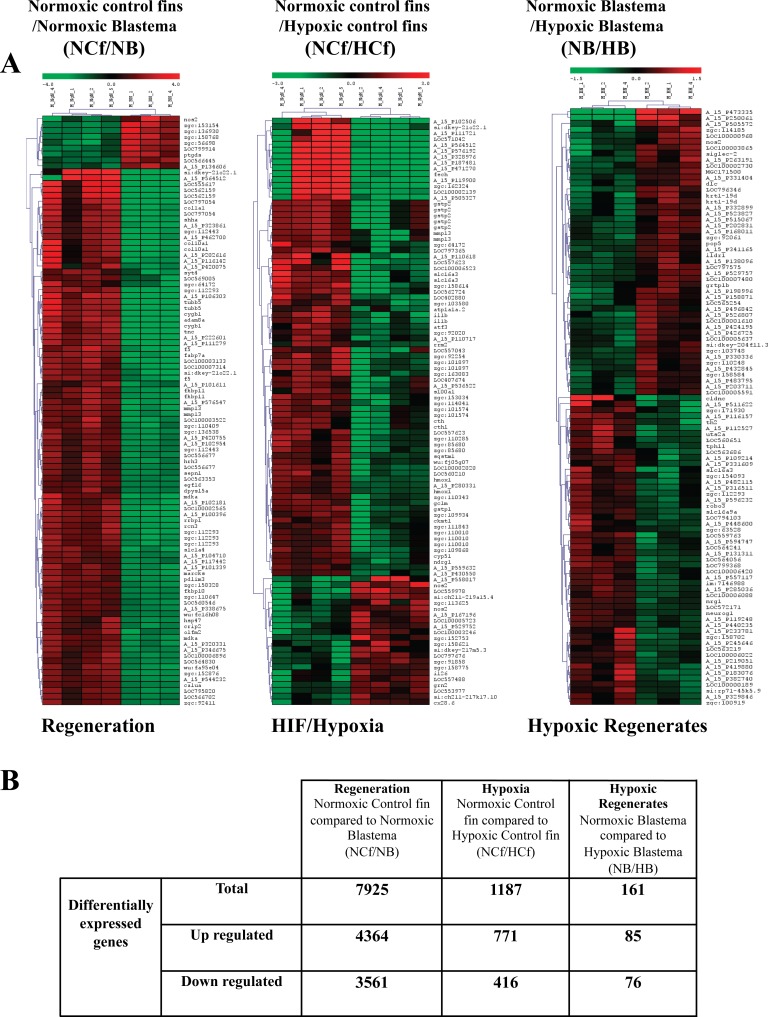
Analysis of differentially regulated genes during regeneration and/or HIF induction **A.** Cluster and heat map analysis. Genes that were differentially expressed during regeneration under normoxic control conditions were obtained through comparison of genes expressed in normoxic control fins (NCf) and genes expressed in normoxic control regenerated area (blastema) (NB) after 3 dpa. Genes that were differentially expressed during HIF induction were derived from the comparison of genes expressed in normoxic control fins (NCf) and genes expressed in CoCl_2_-treated control fins (HCf). Genes that were differentially expressed in CoCl_2_ regenerates were obtained through comparison of genes expressed in control normoxic blastema (NB) and genes expressed in CoCl_2_-treated blastema (HB). The heat map indicates the level of genes expression and expressed as log_2_ (green is a decrease and red an increase relative to control). The color scale is shown at the top. **B.** Summary of number of up-regulated and down-regulated genes during regeneration, HIF induction or both obtained by microarray analysis.

In order to probe the observed impact of HIF on regenerative processes, RNA derived from regenerates of 3 dpa were subjected to global gene expression profiling. Comparison of the resultant expression profiles revealed that a total of 7925 transcripts were differentially expressed during regeneration, as indicated by the comparison of deregulated expressed genes in normoxic control fins (NCf) and normoxic blastemas (regenerated areas) (NB). 1187 genes were differentially expressed during HIF induction as deduced from the comparison of deregulated expressed genes in normoxic control fins (NCf) and CoCl_2_-treated fins (HCf). 161 transcripts were differentially expressed during regeneration in the presence of CoCl_2_, as revealed through comparison of deregulated expressed genes in control normoxic blastemas (NB) and in CoCl_2_ treated-blastemas (HB) (Figure 4A, 4B).

### Functional enrichment of differentially expressed genes

The differentially expressed genes were partitioned into up or down regulated group prior submission to DAVID software and perform functional enrichment analysis. The function clusters in each group are summarized in Table 1 and Table 2. The corresponding enriched genes are presented in Supplemental Table 1. The function clusters specific to up-regulated genes during normoxic regeneration (NCf/NB up) were mainly related to DNA replication and repair, cell migration, morphogenesis and differentiation (Table 2). Up-regulated genes during HIF induction (NCf/HCf up) were mainly linked to stress response, cellular homeostasis and lipid metabolism (Table 2). Further analysis revealed that the major function clusters genes common to NCf/NB up and NCf/HCf up were related to DNA replication, cytoskeleton and proteasome (Tables 1, 3).

**Table 1:** Gene clusters of differentially regulated genes during regeneration, HIF induction or combined HIF induction and regeneration, as deduced by DAVID database analysis

	Differentially regulated genes	Number of genes	Number of clusters (with High score)	Main function or functional enrichment
**Unique to Regeneration (NCf/NB)**	NCf/NB up	3884	19	DNA replication, Cytoskeleton, Extracellular region, Response to DNA Damage stimulus, Fin development, Tissue morphogenesis, Cell motion, EF-HAND 1, small nuclear ribonucleoprotein particles (Sm)
	NCf/NB down	3277	10	Pleckstrin homology, Bromodomain, Intermediate filament, Serine/threonine protein kinase, Cytoskeleton, GTPase regulator activity,
**Unique to** **HIF** **(NCf/HCf)**	NCf/HCf up	291	7	Stress response, Lipid biosynthetic process, Cellular homeostasis
	NCf/HCf down	132	1	MHC protein complex
**Common to regeneration and HIF** **(NCf/NB) and (NCf/HCf)**	NCf/NB and NCf/HCf Up	480	10	Proteasome complex, DNA replication, ATPase, Cell cycle, Cytoskeleton,
	NCf/NB and NCf/HCf Down	284	2 (>2) 5 (>1.5)	Pleckstrin homology-type, Src homology-3 domain, MHC protein complex, Protein kinase activity GTPase regulator activity
**Unique to HIF and regeneration** **(NB/HB)**	NB/HB Up	64	None	Negative regulation of cell differentiation, Negative regulation of cell communication, Metal ion binding
	NB/HB Down	64	None	Immunoglobulin subtype, Notch signaling pathway, Intermediate filament cytoskeleton,

“up”: up-regulated; “down”: down-regulated

**Table 2:** Function enrichment of up-regulated and down-regulated genes involved in biological process during regeneration and HIF induction, as deduced by DAVID database analysis

Regeneration			
Function enrichment of upregulated genes	Enrichment Score	Number of implicated genes	*P* value
small nuclear ribonucleoprotein particles (sm)	6.9	13	5.2 10^−8^
Dna replication, Cytoskeleton	4.94 4.45	23 72	1.4 10^−5^ 1.9 10^−4^
Extracellular region	3.39	86	9.10^−3^
Response to DNA damage stimulus	2.85	26	8.1 10^−3^
Fin development	2.66	20	3.210^−5^
Tissue morphogenesis	2.09	31	9.410^−5^
Cell motion	2.09	38	2.210^−3^
EF-HAND 1	2.03	43	1.110^−3^
			
**Function enrichment of down regulated genes**	**Enrichment Score**	**Number of implicated genes**	***P* value**
Pleckstrin homology	4.11	29	3.10^−5^
Bromodomain	3.15	10	30110^−4^
Intermediate filament	2.82	13	6.910^−9^
Serine/threonine protein kinase	2.79	29	6.310^−3^
Cytoskeleton	2.15	42	2.610^−4^
GTPase regulator activity	2015	27	3.510^−4^

HIF induction			
Function enrichment of up regulated genes	Enrichment Score	Number of implicated genes	*P* value
Stress response	3.48	6	1.310^−5^
Lipid biosynthetic process	2.68	9	2.910^−6^
Cellular homeostasis	2.34	7	6.610^−4^

The clusters are ordered by group enrichment score.

For the down-regulated genes, the function clusters specific to regeneration (NCf/NB down) were related to the cytoskeleton, GTPase and kinase activity and protein-protein interactions (pleckstrin, bromodomain) (Table 2). Following HIF induction, the specific downegulated genes (NCf/HCf down) were regrouped only under function clusters linked MHC protein complex (Table 1). The function clusters that were common to NCf/NB down and NCf/HCf down were linked to protein-protein interactions, GTPase and kinase activity (pleckstrin, Src domain) and MHC protein complex (Table 3).

### Differentially expressed genes specific to regeneration during HIF induction

**Figure 5:**
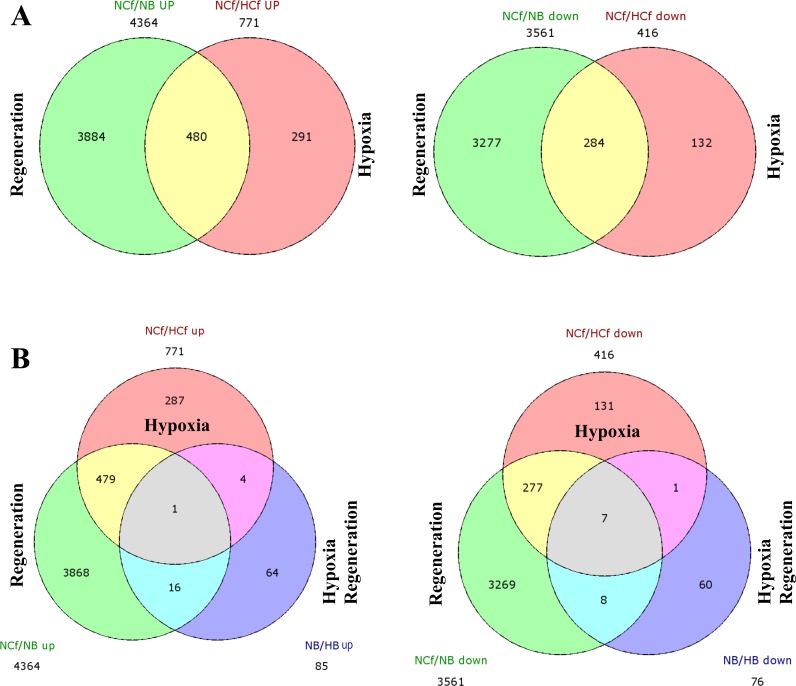
Overlap between regeneration and HIF-related genes After identifying the profiles of differentially expressed genes observed for each comparison (normoxic control fins and regenerated fins (NCf/NB), control fins and CoCl_2_ control fins (NCf/HCf) and regenerated fin and CoCl_2_ regenerated fins (NB/HB), a Venn diagram was performed and revealed similarities and differences in differential transcriptome profiles regulated by regeneration and CoCl_2_ **A.** or by combined regenerative processes and HIF induction **B.** “up” and “down” indicate the up-regulated and down regulated expressed genes, respectively. The number of differentially expressed genes under each condition is also mentioned.

We next analyzed the functional enrichment of differentially expressed genes specific to HIF induction during regeneration as obtained through Venn diagram (Figure 5, Table 1 and Supplementary Table 1). Of the 161 genes differentially regulated during HIF induction and regeneration (control blastemas/CoCl_2_-blastemas (NB/HB), 85 genes were up-regulated with specific expression of 64 genes in NB/HB up and 76 genes were down-regulated with specific 60 genes in NB/HB down. Of the up regulated genes some are involved in the negative regulation of cell differentiation and communication, including neurogenin-1, noggin and phosphatase and tensin homolog A. Accordingly genes involved in positive cell differentiation were down regulated, including delta C and hairy-related 4.1, all involved in Notch signaling pathway (Table 1 and Supplementary Table 1).

### Induction of HIF-related gene targets during normoxic regeneration

In order to identify potential HIF gene targets induced during regenerative angiogenesis, we compared the list of genes differentially expressed during fin regeneration to the list of genes previously reported to be human HIF-related targets [24, 25]. Previously, 7704 genes were identified as HIF binding targets [24, 25]. After conversion of zebrafish genes to their corresponding human orthologs using the conversion tools (http://biodbnet.abcc.ncifcrf.gov/db/dbOrtho.php, http://david.abcc.ncifcrf.gov) we identified a total of 76 genes induced during regeneration that were previously reported to be HIF-associated gene targets in human cells, suggesting that regenerative process controls various HIF-related gene expressions (Supplemental Table 2).

**Table 3:** Function enrichment of up-regulated and down-regulated genes involved in biological processes during HIF induction in regenerated fins, as deduced by DAVID database analysis

**Function enrichment of up-regulated genes**	**Enrichment Score**	**Number of implicated genes**	***P* value**
Proteasome complex	11.2	19	3.9 10-19
DNA replication	9.26	16	4.12 10-12
ATPase,	8.06	18	7.8 10-13
Cell cycle	6.35	24	1.6 10-12
Cytoskeleton	2.36	12	4.02 10-3
**Function enrichment of down-regulated genes**	**Enrichment Score**	**Number of implicated Genes**	***P* value**
Pleckstrin homology-type	2.25	7	3.210^−3^
Src homology-3 domain	2.13	7	1.510^−3^
MHC protein complex	2.13	5	4.10^−4^
Protein kinase activity	1.66	11	1.810^−3^
GTPase regulator activity	1.52	5	2.410^−2^

The clusters are ordered by group enrichment score.

### HIF-dependent and HIF-independent gene targets during HIF/hypoxia and hypoxic regeneration

**Figure 6:**
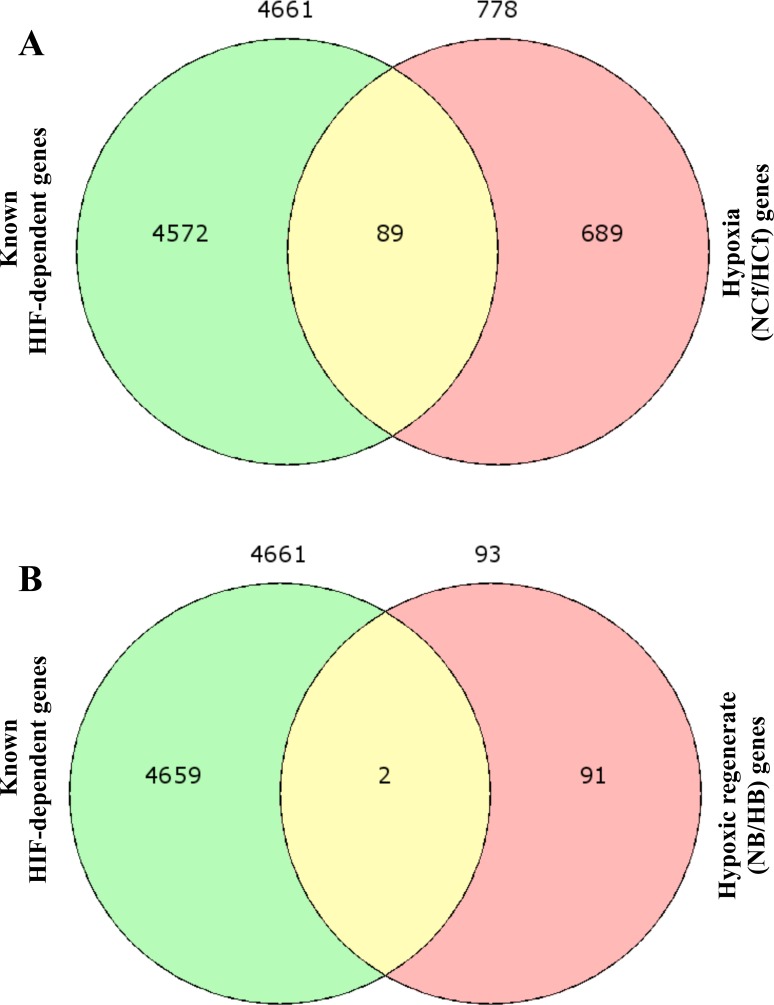
HIF-dependent and HIF-independent gene targets during HIF/hypoxia (NCf/HCf) and hypoxic regeneration (NB/HB) Following the conversion of known human HIF-dependent gene targets to their corresponding zebrafish orthologs using biodbnet conversion tools and the list of the differentially regulated genes during hypoxia and in hypoxic regenerates, Venn diagram identify 89 HIF-dependent and 689 HIF-independent target genes in NCf/HCf **A.** In NB/HB 2 HIF-dependent and 91 HIF-independent gene targets were identified **B.**

Following the conversion of human genes to their corresponding zebrafish orthologs using biodbnet conversion tools, Venn diagram identify 89 HIF-dependent and 689 HIF-independent target genes within the differentially expressed genes in NCf/HCf (Figure 6A, Table 4 and Supplemental Table 3). In hypoxic regenerates 2 HIF-dependent and 91 HIF-independent gene targets were identified (Figure 6B, Table 4 and Supplemental Table 3).

**Table 4:** HIF binding target genes induced during HIF/hypoxia and HIF/hypoxic regeneration deduced from known human HIF target genes

	HIF/Hypoxia	Hypoxic regenerates
HIF dependent genes	abhd4, apex1, atad1b, atad3b, atf3, carhsp1,ccnb2, cdk2, chchd2, ciapin1, cldnh, cyp51,ddx18, ddx56, dhcr7, dhfr, dnajc21, dtl, ect2,elovl6, fdps, gclc, glrx, hey1, hk1, hmgcs1, hmmr, hspa9, hspb1, htatip2, kpna2, maff, mao, mcm4,mcm5, mcrs1, msh2,mt2,mthfd2,nek2,nfil3,nr1d1,nr4a3,pcna,pdp2,pgd,phb,pitpnb,plk1,pmm2,pola2,pold1,pole2,ppih,prdx6,psmb1,psmc2,psmc3,psmc4,psmd13,psmd7,psme3,rad51,rcc1,rdh12,rnaseh2a,rpa2,rrm2,s100a1,sgk1,slc33a1,slc35e1,smc2,spry2,ssb,stat3,thoc6,tk1,trabd,trip13,twistnb,txnl1,tyms,uba1,uhrf1,wars,wee1,zgc:123096,zmpste24.	Cldnc, has2

### Human orthologous genes of the differentially expressed genes during fin regeneration, HIF/hypoxia and HIF/hypoxic regeneration

To list human orthologous genes, the differentially expressed zebrafish genes during regeneration (NCf/NB), HIF induction (NCf/HCf) or HIF/hypoxic regeneration (NB/HB) were converted to their corresponding human orthologous genes using biodbnet conversion tools. These genes are listed in the Supplemental Table 4.

### Effect of col10a1, tubulin, leptin b, cldnc and has2 expression blockade on fin regeneration

**Figure 7:**
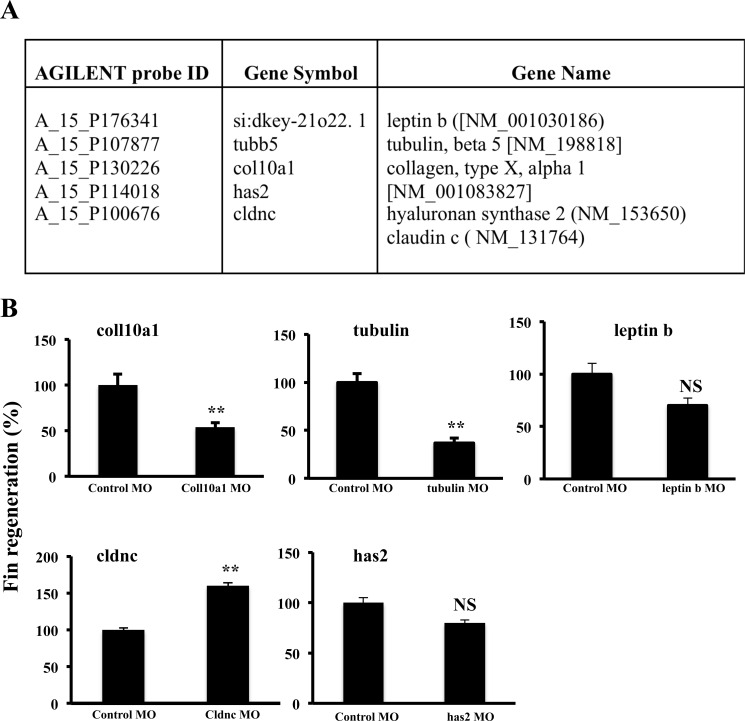
Effect of morpholino (MO) knockdown during zebrafish caudal fin regeneration of col10a1, tubulin, leptin b, cldnc and has 2 genes Caudal fins of adult zebrafish were electroporated with control MO or MOs directed against indicated genes; found to be up-regulated during fin regeneration and/or hypoxic regeneration **A.** and the distal parts of the fins were sectioned and allowed to regenerate for 3 days **B.** Information on the Agilent probe ID, gene symbols and accession numbers are also indicated. Data shown represents Mean ± S.E (*n=*15 per group) from three independent experiments. NS: not significant. **P <0.001.

To evaluate the importance of some of the differentially expressed genes on fin regeneration we assessed the effect of morpholino (MO) knockdown during zebrafish caudal fin regeneration of indicated differentially expressed genes (Figure 7A). Caudal fins of adult zebrafish were electroporated with MOs directed against these genes, and the distal part of the fins was sectioned and allowed to regenerate for 3 days. As illustrated in Figure 7B, fin regeneration was delayed in caudal fins electroporated with col10a1 and tubulin MO compared with caudal fins transfected with control MO. Under the same conditions, caudal fins of zebrafish transfected with leptin b or has2 MO showed only weak effect and repression of cldnc expression induced fin regeneration (Figure 7B).

## DISCUSSION

Analysis of vessel structure during fin regeneration revealed that under control conditions, regenerating fins progressively ceased plexus formation and form vessels with U-shaped connections at the tip of each fin ray. Exposure to CoCl_2_ inhibited this process, and maintained vascular plexuses formation at the distal tips of regenerating fins (Figure 2). This phenomenon was associated with increased numbers of Tip cells, indicators of undifferentiated endothelial cells [26], suggesting that chronic HIF induction to inhibit and/or to retard differentiation of endothelial cells, and to prevent blood vessels to form U-shaped connections adequately. In agreement with this finding, neuroretina-specific knockout of HIF-1α showed impaired vascular development characterized by reduced vessel branching [27]. Similarly, during endochondral ossification, chondrocytes were reported to stop proliferation, exit cell cycle, and differentiate into hypertrophic chondrocytes, which produce collagen-10 and mineralize their surrounding matrix [23, 28]. We found that although HIF induction during fin regeneration was associated with increased collagen-10 expression (Figure 3D), the regenerated fins lacked endochondral ossification (Figure 3B, 3C). Further analysis, revealed that chondrogenesis activity was maintained in the CoCl_2_-treated regenerates leading ultimately to the formation of fragile, easily breakable fins (Figure 3A). In agreement with this finding, previous studies reported that HIF is necessary for the proliferation and survival of chondrocytes in the cartilaginous mould [28]. Bone ossification is characterized by replacement of pre-existing connective tissue by calcified bone matrix. This matrix is formed through extracellular mineralization of osteoid deposited by osteoblasts [23, 28]. During this process, called endochondral ossification, the preformed cartilaginous mould serves as a primary connective tissue. As the cartilage template takes shape, chondrocytes undergo their proliferation prior to their maturation into hypertrophic cells and subsequently apoptosis, while being replaced by bone. Angiogenesis was found temporally to precede osteogenesis since the latter takes place near the newly formed vessels that mediate delivery of osteoprogenitor cells, nutrients and oxygen [29-32]. Previously inadequate bone vascularity was associated with decreased bone formation and bone mass [30, 31], while inhibition of angiogenesis during fracture repair resulted in the formation of fibrous tissue. Thereby, a poor blood supply is therefore considered as a risk factor for an impaired bone healing [30-33].

HIF has been well established as one of the most essential transcriptional factors for adaptation against hypoxia. HIF transcribes various essential genes linked to tissue repair after hypoxic injury, including cell proliferation, apoptosis, energy preservation and angiogenesis [29, 34]. Using DAVID Functional Annotation Clustering Tool we mapped all genes and group of genes exclusively regulated during fin regeneration, HIF induction and during the combination of these processes. Our findings (Table 1 and Supplementary Table 1) indicate that regeneration involves genes required for cytoskeletal organization (lamin A, myosin 1b-like 2, thymosin, beta and catenin), cell proliferation (proliferating cell nuclear antigen and thymidine kinase 1), and cell motility (matrix metalloproteinase 14 and dead end). Various genes involved in cell lineage specification and organ formation were also induced, including connexin 43, exostoses (multiple) 2, and sonic hedgehog-like; sonic hedgehog a and bone morphogenetic protein 4); confirming that during regeneration the main induced transcriptional network is required for tissue development and organogenesis. HIF induction was found to regulate the expression of genes associated with stress response (heat shock proteins −4, −8, −10, −70 and −90), cellular homeostasis (transferrin receptor 1b, glutamate-cysteine ligase and catalytic subunit), lipid biosynthetic process (cholesterol 25-hydroxylase and 7-dehydrocholesterol reductase), and MHC protein complex (major histocompatibility complex class I UEA gene). Kinesin KIF11, which encodes for the Eg5 protein was also expressed under these conditions. The latter was found to be induced by VEGF-A and implicated in zebrafish embryo blood vessel formation [35]. Further analysis indicated that combined together, regeneration and HIF affect the expression of 764 common genes mainly involved in DNA replication (DNA polymerase and retinoblastoma binding protein 4), proteasome complex (proteasome activator subunit 3, proteasome (prosome, macropain) subunit, alpha type, 6a) and cytoskeleton (kinesin family member 14 and HAUS augmin-like complex, subunit 6). These findings suggest that HIF and regeneration control the cell cycle through multiple targets, which can both activate and repress genes that participate in cell cycle regulation. On the other hand, the presence of CoCl2 during fin regeneration was found to induce the up regulation and down regulation of exclusive genes (Figure 4, Table 1 and Supplementary Table 1), mainly involved in cell differentiation and communication. Of the upregulated genes, some are directly implicated in the negative regulation of cell differentiation and communication, including neurogenin 1, noggin 2 and phosphatase and tensin homolog A. Of the down regulated genes, some are linked to notch signaling pathway, such as deltaC and hairy-related 4.1-like, known to provide effective communication between adjacent cells and regulates cell fate decisions, cellular development, and differentiation [36]. Previously, other functions were also attributed to notch signaling pathway such as myocardial regeneration and angiogenesis [37-40]. In our model, the identified negative effect of HIF on key genes and signal pathways involved in cell differentiation is consistent with the undifferentiated vessels in the regenerates treated with CoCl2. In agreement with this finding hypoxia induction was reported to inhibit differentiation of various cells, including mesenchymal cells, and promote dedifferentiation of other cells [40, 41].

In the current study, we only analyzed gene expression changes at 3-dpa intervals. Thus, further studies to evaluate temporal gene expression of selected genes with biological relevance should be carried out to confirm which changes are transient or durable. We found this time interval relevant based on previous studies showing that the peak expression of several genes involved in vessel fin regeneration such VEGF-C and furin occurs around 3-6 dpa [19], thereby providing reasonable evidence that this time point should be the best to compare changes during regeneration and/or HIF/hypoxia. In addition, the formation of vascular plexus in regenerating fins appears only between 3 and 6 days post-amputation [18] indicating that the major transcriptional changes occur mainly close to 3 dpa. Finally, we found that this time interval as optimal for the collection of the minimum regenerated tissues sufficient for the analysis. The functional enrichment analysis used here was helpful to identify possible relevant genes and pathways. A limitation of the present study is that we were only able to detect pathways whose genes are regulated by transcriptional activity. However, other relevant targets that are not regulated at the transcriptional level may have not been disclosed. These targets could be possibly regulated metabolically by HIF and/or regeneration.

In our model, CoCl_2_ induced significantly HIF-1 and HIF-2 expression in control fins while compared to regenerated fin areas at 3 dpa, suggesting the implication of the regenerative processes in the observed reduced HIF expression in the presence of CoCl_2_ (Figure 1). Indeed, previous studies reported that HIF was found to negatively regulate its own expression through downstream effectors involved in the destabilization of HIF mRNA [42]. Thereby, HIF induction during fin regeneration seemed to reduce HIF accumulation induced by CoCl_2_. We also found that regenerative process is able to induce HIF expression under normoxic conditions that was associated with the expression of various genes reported to be HIF-related genes (Supplemental Table 2). The mechanism that controls this process and their biological signification remain unknown.

Although CoCl_2_ is a well-known hypoxia mimetic agent, this water-soluble compound was traditionally used to treat anemia in pregnant women, infants and patients with chronic anemia undergoing long-term hemodialysis [43]. Indeed, Cobalt is a relatively rare transition metal with properties similar to those of iron, chromium, and nickel; thereby it can replace the iron in heme [44] and be a substrate for ferrochelatase, the enzyme responsible for the incorporation of iron into protoporphyrin IX to make heme [45]. The cobalt was also reported to behave as an oxidative stress-inducing factor producing ROS *via* a Fenton-type reaction [46] and act as a pro-oxidant, inducing reactive oxygen species (ROS) levels increase that mediates apoptosis in several cells [47]. In conclusion, in our model, by allowing fin vessel regeneration in the presence of CoCl_2_, we have uncovered the ability of HIF induction in maintaining immature vessels during regenerative angiogenesis. The regenerated fins lacked completely bone formation and generated fragile fins. This is the only model in which limited regeneration and tissue differentiation has occurred under chronic HIF induction. The functional transcriptional analysis conducted in this work allowed the possible detection of new targets, biological process and signaling pathways associated with HIF and regeneration or with both biological processes. Indeed, the use of morpholino knockdown strategy during fin regeneration to repress the expression of some of these genes such as leptin b, tubulin, col10a1, has2 and cldnc (Figure 7A) found to be induced during fin regeneration and/or hypoxic regeneration, revealed that while tubulin and col10a1 repression delayed fin regeneration, leptin b and has2 repression showed weak effect and cldnc repression induced fin regeneration (Figure 7B). Further dissection of the molecular mechanisms found here are demanding to gain potential insights into the importance of HIF in vessel regeneration that may lead to the discovery of effective treatments to ischemia-related diseases. Our study could provide a new foundation to open this field of research. We believe that the generation of this gene expression database, useful tool for the current study will also serve to strategies that aim to use HIF/hypoxia-based angiogenesis as a new therapeutic approach for the possible shift into potential clinical trials.

## MATERIALS AND METHODS

### Experimental animal

Wild-type *Danio rerio* and transgenic *Tg (fli1: EGFP)* zebrafish derived from ZIRC fish center (Oregon) were kept in an aquatic holding facility under standard conditions, as previously described [19, 20]. The photoperiod was 10-h dark/14-h light cycle and water temperature was maintained at 28 ± 1°C throughout holding and during experiments. Ethical approval for all animal studies was obtained from the institutional animal care and use committee in accordance with the national advisory committee for laboratory animal research guidelines.

### *In vivo* HIF induction

Adult fish of at least 10 weeks were anesthetized by the addition of 0.6 μM tricaine (ethyl-m-aminobenzoate) to water. 50 % of caudal fins were surgically removed using a scalpel as indicated in Figure 1A. Animals were placed in each experimental chamber for various time periods depending on the experiment. To induce HIF expression, 1mM of cobalt chloride (CoCl_2_) was dissolved in water, as previously described [19]. To avoid potential toxic effects of nitrogenous waste from fish, water was replaced with fresh one containing CoCl_2_ every 24 h.

### Preparation of total RNA and real-time PCR

For RNA analysis in control and zebrafish regenerated fins, total RNA was extracted using an RNeasy kit (Qiagen) following manufacturer's instructions. Real time-PCR was performed on an ABI 7500 Real Time PCR system (Applied Biosystems), using SYBR GreenER ROX mix (Invitrogen), as previously reported [20]. In brief, a mixture of the reaction consists in 20-μl total volume of 2 μl of cDNA, 2 x QuantiTect SYBER Green PCR Master Mix, and 0.5 μM of forward and reverse primers. PCR reaction was performed at 94°C for 15s and at 60°C for 1 min during 40 cycles. Each sample was amplified in triplicate or duplicate and independently repeated at least three times. The primers used in this study were for Collagen 10: CCGCAGTACCAGCCTTACTC and CTGGCAGACCTTCACCATCT, for HIF-1: TTTAGAGGTGAAAGGGTCCAGTGT and GCCCTCTAGAGAACTGCTCAACA, for HIF-2: GAGAGCTGTGCAGTCATGGA and GTCGGTTGTCCGTTCTGATT and for actin, the internal control: CACAGATCATGTTCGAGACCT and AGGGCGTAACCCTCGTAGAT, respectively.

### Microarray experiments and analysis

For microarray analysis, total RNA was extracted from control and regenerated fins in the absence and presence of CoCl_2_ using the NucleoSpin^®^ RNA II kit (Macherey-Nagel, Germany), according to the manufacturer's instructions. Purity of RNA samples was evaluated and RNA integrity was controlled by the Bioanalyzer 2100 technology (Agilent Technologies, USA) and only samples with RIN value above 8.0 were used in microarray experiments. Gene expression profile analysis was performed using Zebrafish Oligo Microarrays V2 (G2519F, Agilent Technologies, USA). Target preparation and hybridization were done according to the manufacturer's instructions. Briefly, 1 μg of total RNA was labeled using the Quick Amp labeling kit (Agilent Technologies, USA), Cy5-CTP for the reference RNA, Cy3-CTP for individual samples. Internal standards were derived from the Two-Color RNA Spike-in Kit (Agilent Technologies, USA). The labeled targets were purified using the RNeasy^®^ Mini Kit (Qiagen) and their quality and quantity were confirmed by spectrophotometry and the Bioanalyzer 2100 technology (Agilent Technologies, USA). Cy3- and Cy5-labeled cRNA targets were mixed and incubated on microarray slides at 65°C for 17 hours. After washing, the slides were scanned using the Microarray Scanner (Agilent Technologies, USA) at 5-μm resolution and at high and low photomultiplier voltages to optimize the dynamic range of image quantification. The data were extracted from these images using the Agilent Feature Extraction v.9.5.3 software. The data were processed by the EASANA analysis system (GenoSplice technology). Arrays were background corrected using minimum method (any intensity which is zero or negative after background subtraction is set equal to half the minimum of the positive corrected intensities for that array), and within-array loess normalization was performed. Finally, *array* data were normalized using quantile normalization. All experiments were performed at least three times. Statistical analyses were performed using Student's *t*-test on the gene signal intensities. The results were considered statistically significant at *p*-values≤0.05 with fold-change ≥1.5. Hierarchical clustering was carried out to cluster among the gene signal intensities and among the samples with Multi Experiment Viewer (MEV) software [21]. Data obtained were used to construct a Venn diagram identifying the common and exclusively expressed genes of each stratum. The data mining of the genes was performed using DAVID (http://david.abcc.ncifcrf.gov) databases. The data discussed in this study have been deposited in NCBI's Gene Expression Omnibus (http://www.ncbi.nlm.nih.gov/geo/) and are accessible through the GEO Series accession numbers: [GenBank: GSE58566, GSE58567, GSE58568 and GSE58569].

### Regenerative angiogenesis assay and measurement of fin regeneration

**For the regenerative angiogenesis assay,** adult *Tg (fli1: EGFP)* zebrafish were anesthetized in 0.6 μM tricaine, and caudal fins were amputated below the second branch points. Fish were incubated in tank water or in tank water containing 1mM CoCl_2_ for 6 days [19]. At the end of the experiments fins were fixed in 4% PFA for 24 hours at 4°C and mounted on glass slides and visualized on fluorescent microscope. The regenerate length is measured as the distance between amputation plane and the tip of the regenerate. The size of the plexus is measured as the distance between the distal end of regenerating vessels and the proximal margin of the plexus, as defined by the presence of distinctive arteries and veins in the proximal regenerates.

### Morpholino experiments

To block the activity of leptin b, tubulin and col10a1 transcripts synthetic morpholino antisense oligonucleotides (MOs) were synthesized (GeneTools, Philomath, OR, USA) to mask the translational start site. MO sequences used in this study are “TCATTGAAGACTTCATATTTCTGCA” for leptinb; “GGATGTGAACAATCTCCCTCATTGT” for tubb5; “CTCGTAGTTCCATTCTCTGAGGTC” for col10a1; and “CTGACCGCTTTATCACATCTCATCT” for has2; “AAGACGCCATGTCTCCAGTCTCTTT” for cldnc. For zebrafish fin regeneration assay, adult fish of at least 10 weeks were anesthetized by addition of 0.6-μM tricaine to water. Caudal fins were amputated at a level proximal to the first bifurcation of the bony rays and MOs were injected into regenerates as described previously [19]. In brief, following injections, 10 consecutive 50-ms electric pulses, at 15 V with a 1-second pause between pulses, were applied *via* a pair of electrode disks (7 mm in diameter). Twenty-four hours post-injection, caudal fins were amputated at a level proximal to the first bifurcation with a scalpel, and fish were returned to a 28.5°C tank. As a control, Gene Tools’ standard vivo control MO “CCTCTTACCTCAGTTACAATTTATA” was used in this study.

### Alizarin red and alcian blue staining

For bone staining, fins were fixed overnight in 4% paraformaldehyde prior to staining with alizarin red. Briefly dehydrated in 70% ethanol, and placed in 1 g/l alizarin red; 0.5% KOH. Fins were destained in 1% KOH, until background stain was lost. In several experiments, bone and cartilage were doubly stained. Fins were fixed in 4% paraformaldehyde and stained in 0.1% solution of alcian blue (Sigma-Aldrich) dissolved in 70% ethanol and 30% glacial acetic acid for 24h. Fins were then rinsed successively in 80%, 50% and 25% ethanol in PBS and followed by rinses in PBS alone. The fins were next destained, and processed for Alzarin red staining.

### Statistical analysis

All data are presented as ± standard error of mean (SE) unless specifically mentioned. Student's *t* test was applied for statistical analysis, as appropriate. *P* values of <0.05 were considered significant.

## SUPPLEMENTARY TABLES








