# Supplementary material for: Biological outcome and mapping of total factor cascades in response to HIF induction during regenerative angiogenesis
Source: Oncotarget. 2016 Feb 25;7(11):12102–20. doi: 10.18632/oncotarget.7728 (PMC4914272; doi:10.18632/oncotarget.7728)
Supplement: Supplementary file 1 [file oncotarget-07-12102-s001.pdf]

SUPPLEMENTAL FILE-1

Biological outcome and mapping of total factor cascades in response to HIF induction during regenerative angiogenesis

Supplemental Table-1

List of genes involved in biological functions indicated in Table-1

Clicking on the corresponding gene can directly download information about the indicated genes and their biological function.

Up-regulated genes during regeneration

Sm

AGILENT_ID, AGILENT_OLIGO_ID	GENE NAME
1529521	LSM11, U7 small nuclear RNA associated
1533180, 1533180	LSM4 homolog, U6 small nuclear RNA associated (S. cerevisiae)
1540531, 1540531	LSM6 homolog, U6 small nuclear RNA associated (S. cerevisiae)
1549075	LSM7 homolog, U6 small nuclear RNA associated (S. cerevisiae)
1523865	hypothetical protein LOC100148068; small nuclear ribonucleoprotein D2 polypeptide
1533921	small nuclear ribonucleoprotein D1 polypeptide
1538175	small nuclear ribonucleoprotein D3 polypeptide
1552462	small nuclear ribonucleoprotein D3 polypeptide, like
1537805	small nuclear ribonucleoprotein polypeptide E
1521584	small nuclear ribonucleoprotein polypeptide F-like
1544934	small nuclear ribonucleoprotein polypeptides B and B1
1528484	zgc:101136
1539146	zgc:103688

dna replication

AGILENT_ID, AGILENT_OLIGO_ID	GENE NAME
1525880	MCM2 minichromosome maintenance deficient 2, mitotin (S. cerevisiae)
1531206, 1531206, 1531206, 1531206	MCM6 minichromosome maintenance deficient 6, mitotin (S. cerevisiae)
1541471	MCM7 minichromosome maintenance deficient 7 (S. cerevisiae)
1548058	RMI1, RecQ mediated genome instability 1, homolog (S. cerevisiae)
1539635, 1539635, 1539635, 1539635	chromatin assembly factor 1, subunit A (p150)
1533585	heat shock cognate 70-kd protein, like; MCM5 minichromosome maintenance deficient 5 (S. cerevisiae); heat shock cognate 70-kd protein; zgc:174006; similar to heat shock protein 8

1531702	minichromosome maintenance complex component 10
1537571, 1537571	origin recognition complex, subunit 3-like (yeast)
1520346	polymerase (DNA directed), alpha 1
1551603, 1551603	polymerase (DNA directed), beta
1537390	polymerase (DNA directed), mu
1545916, 1545916	primase polypeptide 1; hypothetical protein LOC100149101
1532692, 1532692	replication factor C (activator 1) 3
1516069, 1516069, 1516069	replication factor C (activator 1) 5
1524093	replication protein A1
1517223	retinoblastoma binding protein 4, like
1538841	ribonucleotide reductase M1 polypeptide
1551199	si:dkeyp-35b8.5
1547232, 1547232, 1547232	telomeric repeat binding factor (NIMA-interacting) 1
1522736	thymidine kinase 1, soluble
1535472, 1535472, 1535472, 1535472	topoisomerase (DNA) I, like
1535627, 1535627	zgc:110325
1544665, 1544665	zgc:110810

cytoskeleton

AGILENT_ID, AGILENT_OLIGO_ID	GENE NAME
1526352	F-box protein 5
1523593	HAUS augmin-like complex, subunit 6
1529252	SWI/SNF related, matrix associated, actin dependent regulator of chromatin, subfamily c, member 1
1531460	actin related protein 2/3 complex, subunit 5A
1523188	bactin1
1543091, 1543091	catenin (cadherin-associated protein), alpha 2
1536643, 1536643	cell division cycle associated 8
1522842, 1522842	centrosomal protein 76
1533503	dynein light chain 2, like; dynein light chain 2
1533865	dynein, cytoplasmic 2, light intermediate chain 1
1535015, 1535015	dynein, light chain, LC8-type 2
1519130	engulfment and cell motility 1 (ced-12 homolog, C. elegans)
1550496	glial fibrillary acidic protein
1538241	hypothetical protein LOC553303
1549573	hypothetical protein LOC553479
1524030	intraflagellar transport protein 20
1527496, 1527496	intraflagellar transport protein 52
1550041, 1550041	intraflagellar transport protein 57
1550477	keratin 18
1525327	kinesin family member 11
1521490	kinesin family member 15
1545305	kinesin family member 1B
1533277, 1533277	kinesin family member 3A; similar to kinesin-like protein KIF3Abeta
1523020	kinesin family member 7
1528196	kinesin family member C1; kinesin family member C1-like
1531248, 1531248	lamin A
1549178, 1549178, 1549178	lamin B1
1529379	lamin B2
1517686, 1517686	lamin L3
1545130	metallothionein 2; metallothionein; kinesin family member 20A
1530280	myosin 1b-like 2
1522133, 1522133	nucleolar and spindle associated protein 1
1546191	nudE nuclear distribution gene E homolog like 1 (A. nidulans) B
1530355	polo-like kinase 4 (Drosophila)
1529970	profilin 2 like
1519500	protein phosphatase 4 (formerly X), catalytic subunit a
1516506, 1516506	radixin
1547009	sb:cb152
1520161, 1520161	septin 2
1536804	septin 5a
1519211	septin 8a
1524165	si:ch211-61f14.1

1528515	si:dkey-33c12.3
1547768	si:rp71-68n21.13; similar to LOC553340 protein
1532300	similar to Peptidyl-prolyl cis-trans isomerase NIMA-interacting 4 (Rotamase Pin4) (PPIase Pin4) (Parvulin 14) (Par14) (Peptidyl-prolyl cis/trans isomerase EPVH) (hPar14); zgc:110008
1541347	similar to nonmuscle myosin heavy chain; myosin, heavy polypeptide 10, non-muscle
1516729	spindle assembly 6 homolog (C. elegans)
1533827	talin 2
1520907	thymosin, beta
1551673	troponin T2, cardiac
1534237	tubulin cofactor a
1540273	tubulin, alpha 1
1546341	tubulin, alpha 8 like
1532275, 1532275	tubulin, alpha 8 like 2
1540097	tubulin, alpha 8 like 4; tubulin, alpha 8 like 3; similar to tubulin, alpha 8 like 3; hypothetical LOC791753
1545949, 1545949	tubulin, beta 5
1524356	tubulin, epsilon 1
1525264	tubulin, gamma complex associated protein 2
1550299	tubulin, gamma complex associated protein 3
1517459	tubulin, gamma complex associated protein 4
1544958, 1544958	tubulin, gamma-like
1531086	zgc:101095
1539624	zgc:112515; hypothetical LOC792245
1538539, 1538539	zgc:123298; hypothetical protein LOC100148745; similar to alpha-tubulin isotype M-alpha-2; wu:fb37a10
1530811	zgc:136902
1520894	zgc:153629
1522899	zgc:171223
1539482, 1539482, 1539482	zgc:55461; zgc:123194; zgc:153264; zgc:123292; tubulin, beta 2c; zgc:153426
1533425	zgc:55995
1552419	zgc:56317
1525403	zgc:66125
1544744	zgc:92713; similar to B9 domain-containing protein 2

extracellular region

AGILENT_ID, AGILENT_OLIGO_ID	GENE NAME
1529637	BMP binding endothelial regulator
1543587	GLI pathogenesis-related 2, like
1542287	HtrA serine peptidase 1
1542802	Kallmann syndrome 1a sequence
1522891	Kallmann syndrome 1b sequence
1520367	Kazal-type serine peptidase inhibitor domain 2
1552322	acid phosphatase-like 2
1541735, 1541735	arginyl aminopeptidase (aminopeptidase B)
1549599	bone morphogenetic protein 4
1533114	calumenin a
1531196	calumenin b
1532475	cat eye syndrome chromosome region, candidate 1a; hypothetical LOC100000645
1520195	cat eye syndrome chromosome region, candidate 1b
1525021	coiled-coil domain containing 80
1531335, 1531335, 1531335	collagen type XI alpha-2
1528080, 1528080, 1528080, 1528080	collagen type XVIII, alpha 1
1546442, 1546442	collagen, type I, alpha 1
1543597, 1543597, 1543597, 1543597	collagen, type I, alpha 2
1530627	collagen, type I, alpha 3
1541007, 1541007	collagen, type IV, alpha 5 (Alport syndrome)
1550951	collagen, type IV, alpha 6
1531016	cysteine-rich with EGF-like domains 2
1533729	decapentaplegic and Vg-related 1
1539137	dickkopf 1

1536746	dickkopf homolog 3 (Xenopus laevis)
1527640	fibroblast growth factor 3
1547237, 1547237	fibronectin 1
1533206, 1533206	fibronectin 1b
1536217	follistatin-like 1a
1538320, 1538320	growth differentiation factor 6a
1546928, 1546928	heat shock protein 5
1534528	hemopexin
1524723, 1524723	inhibin, beta Aa
1544957	inhibin, beta Ab
1537494	laminin, alpha 1
1549538	laminin, alpha 5
1537071, 1537071	laminin, beta 1
1533203, 1533203, 1533203	laminin, gamma 1
1533300	matrix metallopeptidase 23a, like
1549497	matrix metalloproteinase 14 (membrane-inserted) alpha
1522686	matrix metalloproteinase 14 (membrane-inserted) beta
1547797	matrix metalloproteinase 2
1536908	matrix metalloproteinase 9
1536282, 1536282	meteorin, glial cell differentiation regulator-like
1522634, 1522634	microfibrillar-associated protein 2
1542093, 1542093	midkine-related growth factor b
1522565	nerve growth factor (beta polypeptide)
1531736	neuroblastoma, suppression of tumorigenicity 1
1518465	noggin 1
1528108	novel protein similar to vertebrate natriuretic peptide precursor C (NPPC)
1516129	ntl-dependent gene 5
1539289, 1539289	olfactomedin-like 3
1521463, 1521463	secreted acidic cysteine rich glycoprotein
1524629	semaphorin 3d
1517209	serum amyloid A-like 1
1546512, 1546512, 1546512	si:busm1-167c3.3; collagen, type V, alpha 2-like
1519112	signal peptide, CUB domain, EGF-like 2
1539427	similar to C3 and PZP-like, alpha-2-macroglobulin domain containing 8; similar to C3 and PZP-like alpha-2-macroglobulin domain-containing protein 8
1547608	similar to LOC494988 protein
1535770	similar to collagen type XXVII proalpha 1 chain
1542579	somatostatin 1
1519007, 1519007	sonic hedgehog-like; sonic hedgehog a
1517175, 1517175	spondin 1b; similar to spondin 1b
1548142	spondin 2a, extracellular matrix protein
1534147	stanniocalcin 1
1522745, 1522745	tetraspanin 18a
1543701, 1543701	tetraspanin 18b
1539215, 1539215	thrombospondin 1
1527000	thrombospondin 3a
1530865, 1530865	thrombospondin 4b
1552582	tissue factor pathway inhibitor a
1546387	tissue inhibitor of metalloproteinase 2; similar to Tissue inhibitor of metalloproteinase 2
1517856	tissue inhibitor of metalloproteinase 2b
1519071	urotensin 2, alpha
1526021	wingless-type MMTV integration site family, member 3
1542465	wingless-type MMTV integration site family, member 5b
1533204	zgc:110124
1536898	zgc:110677
1523257, 1523257	zgc:112443
1529558	zgc:153027
1540650	zgc:158335
1536335	zgc:158671
1518588, 1518588	zgc:172061
1544488, 1544488	zgc:194131; apolipoprotein Eb
1527540	zgc:56201
1525611	zgc:63947

response to DNA damage stimulus

AGILENT_ID, AGILENT_OLIGO_ID	GENE NAME
1522066	APEX nuclease (multifunctional DNA repair enzyme) 1
1529281, 1529281	CHK1 checkpoint homolog (S. pombe)
1546060	CHK2 checkpoint homolog (S. pombe)
1531707	Fanconi anemia, complementation group C
1530714, 1530714	MUS81 endonuclease homolog (yeast)
1543366	X-ray repair complementing defective repair in Chinese hamster cells 4
1552568	X-ray repair complementing defective repair in Chinese hamster cells 5; hypothetical protein LOC100148606
1519516	X-ray repair complementing defective repair in Chinese hamster cells 6
1539635, 1539635, 1539635, 1539635	chromatin assembly factor 1, subunit A (p150)
1519002, 1519002	general transcription factor IIH, polypeptide 2
1548494	general transcription factor IIH, polypeptide 4
1547166	general transcription factor IIH, polypeptide 5
1529072	nei endonuclease VIII-like 3 (E. coli)
1546298	nudix (nucleoside diphosphate linked moiety X)-type motif 1
1518829	pleckstrin homology-like domain, family A, member 3
1551603, 1551603	polymerase (DNA directed), beta
1524228	protein arginine methyltransferase 6
1551199	si:dkeyp-35b8.5
1551979	similar to RAD52 homolog; RAD52 homolog (S. cerevisiae)
1531289	thymine-DNA glycosylase
1522887	uracil-DNA glycosylase
1532050	zgc:110269
1522440	zgc:112496
1536526	zgc:56259
1539212	zgc:91996
1537538	zinc finger, SWIM-type containing 7

fin development

AGILENT_ID, AGILENT_OLIGO_ID	GENE NAME
1544904, 1544904	Bardet-Biedl syndrome 7
1528249, 1528249	PR domain containing 1a, with ZNF domain
1520466	Sec23 homolog A (S. cerevisiae)
1540473, 1540473, 1540473	cadherin 2, neuronal
1522978	connexin 43
1548799, 1548799	exostoses (multiple) 2
1543512	fibroblast growth factor 10a
1526793	fibroblast growth factor 20a
1523613	heat shock 60kD protein 1 (chaperonin)
1545166	homeo box C13b
1524723, 1524723	inhibin, beta Aa
1549538	laminin, alpha 5
1544683, 1544683	muscle segment homeobox B
1548930	sal-like 1a (Drosophila)
1525342, 1525342	sal-like 4 (Drosophila)
1552348, 1552348	smoothened homolog (Drosophila)
1519007, 1519007	sonic hedgehog-like; sonic hedgehog a
1551649, 1551649, 1551649	sp8 transcription factor-like
1521165	sp9 transcription factor
1542465	wingless-type MMTV integration site family, member 5b

tissue morphogenesis

AGILENT_ID, AGILENT_OLIGO_ID	GENE NAME
1525330	D4, zinc and double PHD fingers, family 3
1529078	ELL associated factor 2
1530677, 1530677	GLI-Kruppel family member GLI2a
1520396	MAD homolog 5 (Drosophila)
1517540, 1517540	PPPDE peptidase domain containing 1
1528024, 1528024	Sp5 transcription factor
1545277	T-cell acute lymphocytic leukemia 1
1523091	Tax1 (human T-cell leukemia virus type I) binding protein 3
1545903	bloody fingers
1549599	bone morphogenetic protein 4
1540473, 1540473, 1540473	cadherin 2, neuronal
1524076	dapper homolog 2, antagonist of beta-catenin (xenopus); hypothetical protein LOC100149774
1533774, 1533774, 1533774	decorin
1518571, 1518571	dishevelled associated activator of morphogenesis 1, like
1547237, 1547237	fibronectin 1
1544266, 1544266, 1544266, 1544266	histone deacetylase 1
1539166	hyaluronan synthase 2; similar to hyaluronan synthase 2
1534361	leucine rich repeat containing 6
1549497	matrix metalloproteinase 14 (membrane-inserted) alpha
1522686	matrix metalloproteinase 14 (membrane-inserted) beta
1550768	meckel syndrome, type 1
1531322, 1531322	midkine-related growth factor
1519836	novel protein similar to vertebrate calcium channel, voltage-dependent, beta 4 subunit (CACNB4, zgc:136550); calcium channel, voltage-dependent, beta 4b subunit
1533885	par-6 partitioning defective 6 homolog gamma B (C. elegans)
1548725, 1548725	prickle-like 1 (Drosophila) a
1552348, 1552348	smoothened homolog (Drosophila)
1521730	snail homolog 1a (Drosophila)
1519007, 1519007	sonic hedgehog-like; sonic hedgehog a
1535083	traf and tnf receptor associated protein
1551673	troponin T2, cardiac
1542465	wingless-type MMTV integration site family, member 5b

cell motion

AGILENT_ID, AGILENT_OLIGO_ID	GENE NAME
1539122	ATPase, Na+/K+ transporting, alpha 1 polypeptide
1529078	ELL associated factor 2
1530677, 1530677	GLI-Kruppel family member GLI2a
1542802	Kallmann syndrome 1a sequence
1521980	LIM homeobox 2
1520396	MAD homolog 5 (Drosophila)
1541826	UDP-Gal:betaGlcNAc beta 1,4- galactosyltransferase, polypeptide 1
1540473, 1540473, 1540473	cadherin 2, neuronal
1547846, 1547846	carbohydrate (chondroitin 4) sulfotransferase 11
1541007, 1541007	collagen, type IV, alpha 5 (Alport syndrome)
1543521, 1543521	collagen, type XIV, alpha 1; SRY-box containing gene 10
1552948	dead end
1539137	dickkopf 1
1543512	fibroblast growth factor 10a
1527640	fibroblast growth factor 3
1522965	frizzled homolog 2
1544266, 1544266, 1544266, 1544266	histone deacetylase 1
1539166	hyaluronan synthase 2; similar to hyaluronan synthase 2
1550041, 1550041	intraflagellar transport protein 57
1519617	lamin B receptor
1537494	laminin, alpha 1
1534361	leucine rich repeat containing 6

1540289	lymphocyte enhancer binding factor 1
1549497	matrix metalloproteinase 14 (membrane-inserted) alpha
1522686	matrix metalloproteinase 14 (membrane-inserted) beta
1550768	meckel syndrome, type 1
1523451, 1523451	neuropilin 2a
1546859, 1546859, 1546859	neuropilin 2b
1529119, 1529119	prickle homolog 1 (Drosophila) b
1548725, 1548725	prickle-like 1 (Drosophila) a
1538393	procollagen-lysine, 2-oxoglutarate 5-dioxygenase 3
1530763	roundabout homolog 1
1519724, 1519724	roundabout homolog 3
1524629	semaphorin 3d
1548795	similar to tenascin C; tenascin C
1527285, 1527285	slit (Drosophila) homolog 2
1521730	snail homolog 1a (Drosophila)
1542465	wingless-type MMTV integration site family, member 5b

AGILENT_ID, AGILENT_OLIGO_ID	GENE NAME	
		**EF-HAND 1**
1525853	DNA (cytosine-5-)-methyltransferase 7	
1544180	EF-hand calcium binding domain 7	
1537295	FK506 binding protein 10	
1525353, 1525353	FK506 binding protein 14	
1522269	FK506 binding protein 7; hypothetical LOC791771	
1547656	FK506 binding protein 9	
1539144	Kv channel interacting protein 1 b	
1534881	NADH dehydrogenase (ubiquinone) 1, alpha/beta subcomplex, 1	
1532774	calbindin 2, (calretinin)	
1552391	calbindin 2, like	
1532760	calcyphosine-like	
1544502, 1544502, 1544502, 1544502	calmodulin 2b, (phosphorylase kinase, delta); calmodulin 2a (phosphorylase kinase, delta); calmodulin 3a (phosphorylase kinase, delta); calmodulin 1b; calmodulin 1a; similar to calmodulin 2; calmodulin 3b (phosphorylase kinase, delta)	
1533114	calumenin a	
1531196	calumenin b	
1529848	carboxypeptidase B1 (tissue)	
1535902	centrin 3	
1550694, 1550694	frequenin homolog b (Drosophila); frequenin homolog a (Drosophila)	
1518442	hypothetical LOC792258; polymerase (RNA) II (DNA directed) polypeptide C	
1533940	im:7147183	
1533277, 1533277	kinesin family member 3A; similar to kinesin-like protein KIF3Abeta	
1519235, 1519235, 1519235, 1519235, 1519235	lysine (K)-specific demethylase 6B	
1531870	met proto-oncogene (hepatocyte growth factor receptor); similar to met proto-oncogene (hepatocyte growth factor receptor)	
1521389	myosin, light polypeptide 9, regulatory; zgc:103467; hypothetical LOC792209	
1521576, 1521576	nucleobindin 2a	
1517519	nucleobindin 2b	
1546841	parvalbumin 7	
1543953, 1543953	protein kinase C substrate 80K-H	
1542795	reticulocalbin 3, EF-hand calcium binding domain	
1521463, 1521463	secreted acidic cysteine rich glycoprotein	
1521711, 1521711, 1521711	si:ch211-160d20.1	
1531419	si:ch211-173p18.9	
1539490	si:dkey-216e9.5; similar to 19.9kD myosin light chain	
1542372, 1542372, 1542372	similar to actinin alpha 4	
1531181	solute carrier family 25 (mitochondrial carrier, Aralar), member 12	
1538849, 1538849	sorcin	
1539215, 1539215	thrombospondin 1	
1521424	zgc:103639	
1529842, 1529842	zgc:110317	
1550876	zgc:110594	
1531258	zgc:162879	
1527106, 1527106	zgc:55262	
1533094	zgc:63695	
1519268	zgc:92169; similar to protein phosphatase 3, regulatory subunit B, alpha	

Down-regulated genes during regeneration

Pleckstrin homology

AGILENT_ID, AGILENT_OLIGO_ID	GENE NAME
1542167, 1542167	CDC42 binding protein kinase beta (DMPK-like)
1524786	GRB2-associated binding protein 1
1523032	IL2-inducible T-cell kinase
1545321	Ras protein-specific guanine nucleotide-releasing factor 2
1531988	Rho GTPase activating protein 12
1541671	Rho guanine nucleotide exchange factor (GEF) 3, like
1546714	SET binding factor 1
1523366	connector enhancer of kinase suppressor of Ras 1
1527672	faciogenital dysplasia
1545712, 1545712	novel protein similar to vertebrate Rho guanine exchange factor (GEF) 16 (ARHGEF16)
1532323, 1532323	oxysterol binding protein-like 7
1536345	phospholipase D1a
1527878	pleckstrin homology domain containing, family F (with FYVE domain) member 1
1525427, 1525427	rho/rac guanine nucleotide exchange factor (GEF) 18
1516354	semaphorin 4e; signal transducing adaptor family member 2a
1521687	si:ch211-175p12.1
1528179	si:ch211-194i18.1
1528334	similar to phospholipase C, gamma 2; si:ch211-260p9.3
1521133, 1521133	zgc:136817
1523259, 1523259	zgc:153779
1535478	zgc:153917; similar to Centaurin-beta-1 (Cnt-b1) (ARFGAP with coiled-coil, ANK repeat and PH domain-containing protein 1) (ACAP1)
1544346, 1544346	zgc:158223; src kinase associated phosphoprotein 2
1527288	zgc:158281
1526991	zgc:161981
1521247	zgc:162874; similar to Testis-expressed sequence 2 protein
1539564	zgc:55604
1519123, 1519123	zgc:56306
1522383	zgc:63749
1540247, 1540247	zgc:92360

Bromodomain

AGILENT_ID, AGILENT_OLIGO_ID	GENE NAME
1538914	K(lysine) acetyltransferase 2B
1537226	SWI/SNF related, matrix associated, actin dependent regulator of chromatin, subfamily a, member 2
1549968	TAF1 RNA polymerase II, TATA box binding protein (TBP)-associated factor
1544681	ash1 (absent, small, or homeotic)-like (Drosophila)
1521137	bromodomain containing 9
1541595	hypothetical protein LOC100001344
1535965, 1535965, 1535965	myeloid/lymphoid or mixed-lineage leukemia (trithorax homolog, Drosophila)
1531477	similar to Brd2b protein; bromodomain containing 2b
1546538, 1546538	wu:fi25h02
1537151	zinc finger, MYND domain containing 11; hypothetical protein LOC100148969

intermediate filament

AGILENT_ID, AGILENT_OLIGO_ID	GENE NAME
1537568, 1537568	desmin
1548912, 1548912	keratin 15; type I cytokeratin, enveloping layer, like; si:dkeyp-113d7.7; type I

	cytokeratin, enveloping layer
1536940, 1536940	keratin 4
1545525	keratin, type 1, gene 19d
1526244	si:dkey-25f3.3
1537977	similar to internexin neuronal intermediate filament protein, alpha; zgc:65851
1541795	type I cytokeratin; zgc:109868; si:dkeyp-113d7.4
1526598, 1526598	vimentin
1537113	wu:fb15e04
1531018	zgc:136930
1520684	zgc:153753
1543352, 1543352	zgc:73275
1543435	zgc:92380

Serine/threonine protein kinase

AGILENT_ID, AGILENT_OLIGO_ID	GENE NAME
1542167, 1542167	CDC42 binding protein kinase beta (DMPK-like)
1523849, 1523849, 1523849	MAP kinase-interacting serine/threonine kinase 2b
1530089	calcium/calmodulin-dependent protein kinase (CaM kinase) II delta 1
1547923	calcium/calmodulin-dependent protein kinase (CaM kinase) II gamma 1
1539048	hypothetical LOC564413
1520226	mitogen-activated protein kinase 14b
1523938	mitogen-activated protein kinase 3
1540426	mitogen-activated protein kinase 7
1546238	mitogen-activated protein kinase kinase kinase kinase 2-like
1546929	pim-1 oncogene
1520690	protein kinase C, beta 1
1535493	protein kinase C, beta 1, like
1516288	protein kinase C, delta
1535244	protein kinase C, eta
1542324	protein kinase C, theta
1534322	protein kinase, cGMP-dependent, type I
1518210	ribosomal protein S6 kinase, like
1517791	ribosomal protein S6 kinase, polypeptide 3a
1539328	serum/glucocorticoid regulated kinase family, member 3
1547978	si:ch211-81a5.7
1528168	si:dkey-261h15.1
1551932	similar to SNF1/AMP-activated protein kinase
1533706, 1533706	zgc:136354
1549238	zgc:136819; similar to Serine/threonine-protein kinase PFTAIRE-2 (Serine/threonine-protein kinase ALS2CR7) (Amyotrophic lateral sclerosis 2 chromosomal region candidate gene 7 protein homolog)
1537791, 1537791	zgc:153020; hypothetical protein LOC100149364
1522337	zgc:153952
1552956	zgc:162290
1552321	zgc:172124
1516388	zgc:63495

cytoskeleton

AGILENT_ID, AGILENT_OLIGO_ID	GENE NAME
1517535	ADP-ribosylation factor-like 2 binding protein
1534277	ARP3 actin-related protein 3 homolog (yeast)
1526586	ELMO/CED-12 domain containing 2
1536391, 1536391	Janus kinase 3 (a protein tyrosine kinase, leukocyte)
1541398	Wiskott-Aldrich syndrome (eczema-thrombocytopenia) b
1547072	actin, alpha 2, smooth muscle, aorta
1548079	amyotrophic lateral sclerosis 2a (juvenile)
1537568, 1537568	desmin
1547472, 1547472	eukaryotic translation initiation factor 4A, isoform 2
1517636	ezrin
1534519	hypothetical protein LOC100149534; zgc:86725
1521293, 1521293	influenza virus NS1A binding protein a

1533824, 1533824	influenza virus NS1A binding protein b; similar to influenza virus NS1A binding protein b
1548912, 1548912	keratin 15; type I cytokeratin, enveloping layer, like; si:dkeyp-113d7.7; type I cytokeratin, enveloping layer
1536940, 1536940	keratin 4
1545525	keratin, type 1, gene 19d
1545172, 1545172	myosin VA (heavy polypeptide 12, myoxin)
1541452	myosin VB
1526038	myosin regulatory light chain interacting protein a
1516699	myosin, heavy polypeptide 11, smooth muscle
1551440, 1551440, 1551440	myosin, heavy polypeptide 9, non-muscle
1551738	nudE nuclear distribution gene E homolog like 1 (A. nidulans) A
1537108	platelet-activating factor acetylhydrolase, isoform Ib, alpha subunit a
1537242, 1537242	protein tyrosine kinase 2 beta, b
1532015	septin 9b
1526244	si:dkey-25f3.3
1534060	si:dkeyp-92c9.3
1537977	similar to internexin neuronal intermediate filament protein, alpha; zgc:65851
1532275	tubulin, alpha 8 like 2
1541795	type I cytokeratin; zgc:109868; si:dkeyp-113d7.4
1526598, 1526598	vimentin
1534412	vinculin
1537113	wu:fb15e04
1552500	zgc:101797
1531018	zgc:136930
1521783	zgc:153436
1520684	zgc:153753
1516629	zgc:66299
1543352, 1543352	zgc:73275
1551413	zgc:86896
1552217	zgc:92107
1543435	zgc:92380

GTPase regulator activity

AGILENT_ID, AGILENT_OLIGO_ID	GENE NAME
1516876	ADP-ribosylation factor GTPase activating protein 3
1542167, 1542167	CDC42 binding protein kinase beta (DMPK-like)
1545321	Ras protein-specific guanine nucleotide-releasing factor 2
1525926	RasGEF domain family, member 1Ba
1543262	RasGEF domain family, member 1Bb
1531996	Rho GDP dissociation inhibitor (GDI) gamma
1541671	Rho guanine nucleotide exchange factor (GEF) 3, like
1541398	Wiskott-Aldrich syndrome (eczema-thrombocytopenia) b
1548079	amyotrophic lateral sclerosis 2a (juvenile)
1527672	faciogenital dysplasia
1546238	mitogen-activated protein kinase kinase kinase kinase 2-like
1545712, 1545712	novel protein similar to vertebrate Rho guanine exchange factor (GEF) 16 (ARHGEF16)
1525427, 1525427	rho/rac guanine nucleotide exchange factor (GEF) 18
1521687	si:ch211-175p12.1
1528179	si:ch211-194i18.1
1552190, 1552190, 1552190	si:dkey-12o15.1
1523633, 1523633	similar to HIV-1 Rev-binding protein-like protein; zgc:114045
1531774	similar to TBC1 domain family, member 17; zgc:110443
1523365	similar to dedicator of cytokinesis 8
1545553	similar to melanophilin b; melanophilin b
1533706, 1533706	zgc:136354
1521133, 1521133	zgc:136817
1523259, 1523259	zgc:153779
1552270, 1552270	zgc:153898
1535478	zgc:153917; similar to Centaurin-beta-1 (Cnt-b1) (ARFGAP with coiled-coil, ANK repeat and PH domain-containing protein 1) (ACAP1)
1526991	zgc:161981
1540247, 1540247	zgc:92360

Up regulated genes during HIF induction

stress response

AGILENT_ID, AGILENT_OLIGO_ID	GENE NAME
1533585, 1533585	heat shock cognate 70-kd protein, like; MCM5 minichromosome maintenance deficient 5 (S. cerevisiae); heat shock cognate 70-kd protein; zgc:174006; similar to heat shock protein 8
1529921	heat shock protein 4, like
1547371	heat shock protein 9
1544881	heat shock protein 90, alpha (cytosolic), class B member 1
1529911	heat shock protein 90-alpha 2
1528938	novel protein similar to vertebrate heat shock 70kDa protein 1B (HSPA1B)

lipid biosynthetic process

AGILENT_ID, AGILENT_OLIGO_ID	GENE NAME
1532046, 1532046	3-hydroxy-3-methylglutaryl-Coenzyme A synthase 1 (soluble)
1547290, 1547290	7-dehydrocholesterol reductase
1538677, 1538677	ELOVL family member 6, elongation of long chain fatty acids (yeast)
1534901, 1534901	NAD(P) dependent steroid dehydrogenase-like
1528715, 1528715	cholesterol 25-hydroxylase
1539272, 1539272	cytochrome b5 reductase 2
1521023	degenerative spermatocyte homolog 2, lipid desaturase (Drosophila)
1540632, 1540632, 1540632	farnesyl diphosphate synthase (farnesyl pyrophosphate synthetase, dimethylallyltranstransferase, geranyltranstransferase)
1550490	zgc:103473

cellular homeostasis

AGILENT_ID, AGILENT_OLIGO_ID	GENE NAME
1528505, 1528505	glutamate-cysteine ligase, catalytic subunit
1529025	hypothetical LOC792204; thioredoxin-like 1
1549157	peroxiredoxin 6; similar to peroxiredoxin 6
1522124	transferrin receptor 1b
1550958	wu:fj24c01
1526777, 1526777, 1526777	zgc:110010
1525793	zgc:110343

Down-regulated genes during HIF induction

MHC protein complex

ENT_ID	AGILENT_OLIGO_ID	GENE NAME
27		major histocompatibility complex class I UEA gene
46		si:busm1-228j01.6; hypothetical LOC777705
22		zgc:123107; HLA-DPA1 protein

Common up-regulated genes to regeneration and HIF induction

proteasome complex

AGILENT_ID, AGILENT_OLIGO_ID	GENE NAME
1550870, 1550870	hypothetical protein LOC100149336; proteasome (prosome, macropain) 26S subunit, non-ATPase, 4
1544960	proteasome (prosome, macropain) 216S subunit, non-ATPase, 11b
1543279	proteasome (prosome, macropain) 26S subunit, ATPase 2
1542885	proteasome (prosome, macropain) 26S subunit, ATPase, 1b
1534585	proteasome (prosome, macropain) 26S subunit, ATPase, 3
1523338	proteasome (prosome, macropain) 26S subunit, ATPase, 4
1532293	proteasome (prosome, macropain) 26S subunit, ATPase, 6
1540407	proteasome (prosome, macropain) 26S subunit, non-ATPase, 13; similar to proteasome (prosome, macropain) 26S subunit, non-ATPase, 13
1540216	proteasome (prosome, macropain) 26S subunit, non-ATPase, 2
1527512	proteasome (prosome, macropain) 26S subunit, non-ATPase, 6
1552935	proteasome (prosome, macropain) 26S subunit, non-ATPase, 7 (Mov34 homolog)
1551124	proteasome (prosome, macropain) subunit, alpha type, 6a
1524827	proteasome (prosome, macropain) subunit, beta type, 1; similar to proteasome (prosome, macropain) subunit, beta type, 1
1541183, 1541183	proteasome (prosome, macropain) subunit, beta type, 4
1546923	proteasome (prosome, macropain) subunit, beta type, 6
1533970	proteasome (prosome, macropain) subunit, beta type, 7
1537722	proteasome activator subunit 3
1517035	si:rp71-45k5.4; proteasome (prosome, macropain) subunit, alpha type, 2
1545189	similar to Proteasome (prosome, macropain) 26S subunit, ATPase, 1a; proteasome (prosome, macropain) 26S subunit, ATPase, 1a

DNA replication

AGILENT_ID, AGILENT_OLIGO_ID	GENE NAME
1535287	DNA polymerase nu; zgc:153228
1552914	GINS complex subunit 2
1524856	MCM3 minichromosome maintenance deficient 3 (S. cerevisiae)
1517733, 1517733	MCM4 minichromosome maintenance deficient 4, mitotin (S. cerevisiae)
1538735	defective in sister chromatid cohesion 1 homolog (S. cerevisiae)
1533585, 1533585	heat shock cognate 70-kd protein, like; MCM5 minichromosome maintenance deficient 5 (S. cerevisiae); heat shock cognate 70-kd protein; zgc:174006; similar to heat shock protein 8
1516592	novel protein similar to vertebrate polymerase (DNA directed), epsilon (POLE)
1529638	origin recognition complex, subunit 6 homolog-like (yeast)
1544436	polymerase (DNA directed), alpha 2
1525590	polymerase (DNA directed), delta 1, catalytic subunit
1537153	polymerase (DNA directed), epsilon 2
1545916, 1545916	primase polypeptide 1; hypothetical protein LOC100149101
1530820, 1530820, 1530820	proliferating cell nuclear antigen
1544161, 1544161	retinoblastoma binding protein 4
1551083, 1551083	ribonucleotide reductase M2 polypeptide
1528456, 1528456	zgc:110727

ATPase, AAA+ type, core

AGILENT_ID, AGILENT_OLIGO_ID	GENE NAME
1529611	ATP-binding cassette, sub-family F (GCN20), member 1
1544230	ATPase family, AAA domain containing 1b
1519803	ATPase family, AAA domain containing 3B
1524856	MCM3 minichromosome maintenance deficient 3 (S. cerevisiae)
1517733, 1517733	MCM4 minichromosome maintenance deficient 4, mitotin (S. cerevisiae)
1535561	PIF1 5'-to-3' DNA helicase homolog (S. cerevisiae)
1516871	RAD51 homolog (RecA homolog, E. coli) (S. cerevisiae)
1537901	RuvB-like 1 (E. coli)
1546016	origin recognition complex, subunit 1-like
1543279	proteasome (prosome, macropain) 26S subunit, ATPase 2
1542885	proteasome (prosome, macropain) 26S subunit, ATPase, 1b
1534585	proteasome (prosome, macropain) 26S subunit, ATPase, 3
1523338	proteasome (prosome, macropain) 26S subunit, ATPase, 4
1532293	proteasome (prosome, macropain) 26S subunit, ATPase, 6
1545189	similar to Proteasome (prosome, macropain) 26S subunit, ATPase, 1a; proteasome (prosome, macropain) 26S subunit, ATPase, 1a
1540124, 1540124	thyroid hormone receptor interactor 13
1535446	torsin family 2, member A
1518406	zgc:174506

cell cycle

AGILENT_ID, AGILENT_OLIGO_ID	GENE NAME
1542188, 1542188	H2A histone family, member X
1523593, 1523593	HAUS augmin-like complex, subunit 6
1536643	cell division cycle associated 8
1530849	chromatin assembly factor 1, subunit B
1536773, 1536773	cyclin B2
1519533	cyclin E2
1543278	cyclin F
1538735	defective in sister chromatid cohesion 1 homolog (S. cerevisiae)
1545047	establishment of cohesion 1 homolog 2 (S. cerevisiae)
1533585, 1533585	heat shock cognate 70-kd protein, like; MCM5 minichromosome maintenance deficient 5 (S. cerevisiae); heat shock cognate 70-kd protein; zgc:174006; similar to heat shock protein 8
1539663, 1539663	pelota homolog (Drosophila)
1535751, 1535751	polo-like kinase 1 (Drosophila)
1544161, 1544161	retinoblastoma binding protein 4
1539496, 1539496	si:ch211-242m18.4
1545000	si:dkeyp-26a9.1
1547768	si:rp71-68n21.13; similar to LOC553340 protein
1516729	spindle assembly 6 homolog (C. elegans)
1539633, 1539633	ttk protein kinase
1517817	zgc:158297
1545773	zgc:158618
1530750	zgc:162965
1543561, 1543561	zgc:56708
1532969, 1532969	zgc:64116
1540818	zgc:92321

cytoskeleton

;ILENT_OLIGO_ID	GENE NAME
1523593, 1523593	HAUS augmin-like complex, subunit 6
1536643	cell division cycle associated 8
1538360	kinesin family member 14
1521490	kinesin family member 15
1521149, 1521149	kinesin family member 23
1545130	metallothionein 2; metallothionein; kinesin family member 20A
1547768	si:rp71-68n21.13; similar to LOC553340 protein
1516729	spindle assembly 6 homolog (C. elegans)

1541795	type I cytokeratin; zgc:109868; si:dkeyp-113d7.4
1530750	zgc:162965
1525403	zgc:66125
1540818	zgc:92321

Common down-regulated genes to regeneration and HIF induction

Pleckstrin homology-type

AGILENT_ID, AGILENT_OLIGO_ID	GENE NAME
1543273	Rho guanine nucleotide exchange factor (GEF) 7a
1535078	dual adaptor of phosphotyrosine and 3-phosphoinositides
1530505	novel protein similar to vertebrate Rho GTPase activating protein 15 (ARHGAP15)
1526823	similar to neurofibromin 2
1528334	similar to phospholipase C, gamma 2; si:ch211-260p9.3
1537797	zgc:153920
1544346	zgc:158223; src kinase associated phosphoprotein 2

Src homology-3 domain

AGILENT_ID, AGILENT_OLIGO_ID	GENE NAME
1543273	Rho guanine nucleotide exchange factor (GEF) 7a
1523155	SAM domain, SH3 domain and nuclear localisation signals, 1b
1548350, 1548350	si:dkey-33i22.2
1526563	zgc:152911
1544346	zgc:158223; src kinase associated phosphoprotein 2
1516966, 1516966	zgc:63672
1548374	zgc:65942

MHC protein complex

AGILENT_ID, AGILENT_OLIGO_ID	GENE NAME
1551203, 1551203	invariant chain-like protein 2
1537646, 1537646	si:busm1-228j01.6; hypothetical LOC777705
1548031	similar to H-2 class II histocompatibility antigen, A-K alpha chain; novel protein with a Class II histocompatibility antigen, alpha domain and a Immunoglobulin C1-set domain
1525933	zgc:64115
1530683, 1530683	zgc:64161

protein kinase activity

AGILENT_ID, AGILENT_OLIGO_ID	GENE NAME
1516140, 1516140	TRAF2 and NCK interacting kinase a
1528469	calcium/calmodulin-dependent protein kinase IV
1519959	si:busm1-122b7.1
1549841	si:ch211-285c6.2; hypothetical LOC569317
1519049	si:ch211-285c6.3
1547679	si:ch211-66i11.1
1548350, 1548350	si:dkey-33i22.2
1543638	similar to aldehyde oxidase 3; aldehyde oxidase 3
1530814	spleen tyrosine kinase
1522337	zgc:153952
1538832	zgc:64054

GTPase regulator activity

AGILENT_ID, AGILENT_OLIGO_ID	GENE NAME
1543273	Rho guanine nucleotide exchange factor (GEF) 7a
1516140, 1516140	TRAF2 and NCK interacting kinase a
1543922	hypothetical LOC791767; growth hormone regulated TBC protein 1b
1530505	novel protein similar to vertebrate Rho GTPase activating protein 15 (ARHGAP15)
1534354	zgc:158411
