# Supplementary material for: Biological outcome and mapping of total factor cascades in response to HIF induction during regenerative angiogenesis
Source: Oncotarget. 2016 Feb 25;7(11):12102–20. doi: 10.18632/oncotarget.7728 (PMC4914272; doi:10.18632/oncotarget.7728)
Supplement: Supplementary file 2 [file oncotarget-07-12102-s002.pdf]

Supplemental Table-2

Human hypoxia-related genes induced during normoxic fin regeneration

AGILENT_ID, AGILENT_OLIGO_ID	DAVID ID	Gene name
**1516592**	A_15_P145576	novel protein similar to vertebrate polymerase (DNA directed), epsilon (POLE)
**1517972**	**A_15_P173496**	acetyl-CoA acetyltransferase 2
**1519122**	A_15_P201331	ankyrin repeat and zinc finger domain containing 1
**1520150**	A_15_P143211	c20orf20 homolog (H. sapiens)
**1520556**	A_15_P113377	EGF-containing fibulin-like extracellular matrix protein 2
**1521060**	A_15_P104547	inhibitor of growth family, member 2
**1522133**	A_15_P103348	nucleolar and spindle associated protein 1
**1522736**	A_15_P112026	thymidine kinase 1, soluble
**1523539**	A_15_P418340	catenin, beta 2; catenin (cadherin-associated protein), beta 1
**1523745**	A_15_P116564	Rac GTPase-activating protein 1
**1523869**	A_15_P107046	dihydrofolate reductase
**1524637**	A_15_P161341	aldehyde dehydrogenase 18 family, member A1
**1525021**	A_15_P116203	coiled-coil domain containing 80
**1525263**	A_15_P187621	PIH1 domain containing 1
**1525327**	A_15_P106965	kinesin family member 11
**1526232**	A_15_P142146	si:dkey-217k21.2
**1526329**	A_15_P364890	myeloblastosis oncogene-like 2
**1526921**	A_15_P116677	Ras association (RalGDS/AF-6) domain family 1
**1527284**	A_15_P108262	testis derived transcript (3 LIM domains)
**1527300**	A_15_P114402	similar to tumor endothelial marker 8
**1527512**	A_15_P100140	proteasome (prosome, macropain) 26S subunit, non-ATPase, 6
**1527661**	A_15_P119814	thymidylate synthase
**1528196**	A_15_P117464	kinesin family member C1; kinesin family member C1-like
**1529102**	A_15_P115077	solute carrier family 35, member E3
**1529222**	A_15_P105131	wu:fa95e03
**1529878**	A_15_P113715	lactate dehydrogenase A4

**1530049**	A_15_P111998	SMT3 suppressor of mif two 3 homolog 3 (S. cerevisiae)
**1530552**	A_15_P147921	nuclear factor, interleukin 3 regulated
**1530660**	A_15_P118305	BTB (POZ) domain containing 6
**1530840**	A_15_P115339	solute carrier family 39 (zinc transporter), member 13
**1531729**	A_15_P110017	carbohydrate (chondroitin) synthase 1
**1531917**	A_15_P115554	transforming, acidic coiled-coil containing protein 3
**1532321**	A_15_P101762	denticleless homolog (Drosophila)
**1532499**	A_15_P109439	runt-related transcription factor 1
**1532912**	A_15_P109316	sprouty-related, EVH1 domain containing 2
**1533232**	A_15_P175776	microtubule associated serine/threonine kinase-like
**1533470**	A_15_P135601	smoothelin
**1533803**	A_15_P120689	procollagen-proline, 2-oxoglutarate 4-dioxygenase (proline 4-hydroxylase), alpha polypeptide I
**1535217**	A_15_P116212	procollagen-proline, 2-oxoglutarate 4-dioxygenase (proline 4-hydroxylase), alpha polypeptide 2; hypothetical protein LOC100151456
**1536142**	A_15_P140096	dihydropyrimidinase-like 2
**1537436**	A_15_P113425	zgc:76940; wu:fi22e08
**1537909**	A_15_P117117	maternal embryonic leucine zipper kinase
**1538921**	A_15_P366695	non imprinted in Prader-Willi/Angelman syndrome 1
**1538929**	A_15_P100851	cyclin G1
**1539062**	A_15_P115848	carbohydrate (N-acetylgalactosamine 4-0) sulfotransferase 14
**1539984**	A_15_P518912	non-SMC condensin II complex, subunit G2
**1540124**	A_15_P106314	thyroid hormone receptor interactor 13
**1540554**	A_15_P115604	solute carrier family 35, member E1
**1540757**	A_15_P105507	spermine synthase
**1541347**	A_15_P185246	similar to nonmuscle myosin heavy chain; myosin, heavy polypeptide 10, non-muscle
**1542783**	A_15_P161756	integrin, beta 5
**1542967**	A_15_P100471	G protein-coupled receptor 161
**1543346**	A_15_P170361	similar to nuclear prelamin A recognition factor; nuclear prelamin A recognition factor
**1544025**	A_15_P304326	ubiquitin-like, containing PHD and RING finger domains, 1

**1545236**	A_15_P117025	THO complex 6 homolog (Drosophila)
**1545744**	A_15_P120167	hexokinase 1
**1545840**	A_15_P101215	potassium channel tetramerisation domain containing 15; potassium channel tetramerisation domain containing 15, like; similar to potassium channel tetramerisation domain containing 1; novel potassium channel tetramerisation domain protein
**1546226**	1546226	AT rich interactive domain 3B (Bright like); hypothetical protein LOC100150689
**1546968**	A_15_P101408	elongation factor, RNA polymerase II, 2
**1547021**	A_15_P100910	solute carrier family 16 (monocarboxylic acid transporters), member 1
**1547141**	A_15_P120888	lipoma HMGIC fusion partner
**1547247**	A_15_P120075	epithelial cell transforming sequence 2 oncogene
**1547749**	A_15_P468085	zgc:77115
**1548054**	A_15_P189841	crystallin, beta B2
**1548549**	A_15_P104979	serine (or cysteine) proteinase inhibitor, clade E (nexin, plasminogen activator inhibitor type 1), member 2; zgc:158605
**1548598**	A_15_P288206	lon peptidase 1, mitochondrial
**1549103**	A_15_P190356	sprouty homolog 1 (Drosophila)
**1550076**	A_15_P102940	transmembrane protein 48
**1550308**	A_15_P104207	procollagen-lysine, 2-oxoglutarate 5-dioxygenase 2
**1551353**	A_15_P141731	myeloid/lymphoid or mixed lineage-leukemia translocation to 3 homolog (Drosophila)
**1552133**	A_15_P148971	lysyl oxidase
**1552242**	A_15_P110083	chromatin licensing and DNA replication factor 1
**1552527**	A_15_P101012	forkhead box D1
**1552882**	A_15_P161876	pyroglutamyl-peptidase I
